# Three mutations switch H7N9 influenza to human-type receptor specificity

**DOI:** 10.1371/journal.ppat.1006390

**Published:** 2017-06-15

**Authors:** Robert P. de Vries, Wenjie Peng, Oliver C. Grant, Andrew J. Thompson, Xueyong Zhu, Kim M. Bouwman, Alba T. Torrents de la Pena, Marielle J. van Breemen, Iresha N. Ambepitiya Wickramasinghe, Cornelis A. M. de Haan, Wenli Yu, Ryan McBride, Rogier W. Sanders, Robert J. Woods, Monique H. Verheije, Ian A. Wilson, James C. Paulson

**Affiliations:** 1 Departments of Molecular Medicine, & Immunology and Microbiology, The Scripps Research Institute, La Jolla, CA, United States of America; 2 Department of Chemical Biology and Drug Discovery, Utrecht Institute for Pharmaceutical Sciences, Utrecht University, CG Utrecht, The Netherlands; 3 Complex Carbohydrate Research Center, University of Georgia, Athens, GA, United States of America; 4 Department of Integrative Structural and Computational Biology, The Scripps Research Institute, La Jolla, CA, United States of America; 5 Pathology Division, Department of Pathobiology, Faculty of Veterinary Medicine, Utrecht University, Yalelaan 1, CL Utrecht, The Netherlands; 6 Department of Medical Microbiology, Academic Medical Center, University of Amsterdam, AZ Amsterdam, The Netherlands; 7 Virology Division, Department of Infectious Diseases & Immunology, Faculty of Veterinary Medicine, Utrecht University, Yalelaan 1,CL Utrecht, The Netherlands; 8 Department of Microbiology and Immunology, Weil Medical College of Cornell University, New York, NY, United States of America; 9 Skaggs Institute for Chemical Biology, The Scripps Research Institute, La Jolla, CA, United States of America; Icahn School of Medicine at Mount Sinai, UNITED STATES

## Abstract

The avian H7N9 influenza outbreak in 2013 resulted from an unprecedented incidence of influenza transmission to humans from infected poultry. The majority of human H7N9 isolates contained a hemagglutinin (HA) mutation (Q226L) that has previously been associated with a switch in receptor specificity from avian-type (NeuAcα2-3Gal) to human-type (NeuAcα2-6Gal), as documented for the avian progenitors of the 1957 (H2N2) and 1968 (H3N2) human influenza pandemic viruses. While this raised concern that the H7N9 virus was adapting to humans, the mutation was not sufficient to switch the receptor specificity of H7N9, and has not resulted in sustained transmission in humans. To determine if the H7 HA was capable of acquiring human-type receptor specificity, we conducted mutation analyses. Remarkably, three amino acid mutations conferred a switch in specificity for human-type receptors that resembled the specificity of the 2009 human H1 pandemic virus, and promoted binding to human trachea epithelial cells.

## Author summary

Influenza A virus of the H7N9 subtype continues to cross the species barrier from poultry to humans. This zoonotic ability is remarkable as the virus retains specificity to avian-type receptors. To effectively transmit between humans, the virus needs to acquire human-type receptor specificity. In this study, we show that recombinant H7 proteins need three amino acid mutations to change specificity to human-type receptors. Although we are not allowed to assess if these mutations would lead to efficient transmission in the ferret model, this knowledge will aid in surveillance. If these amino acid mutations are observed to arise during natural selection in humans, timely actions could be taken.

## Introduction

The 2013 avian H7N9 virus outbreak in China was tied to human exposure to infected poultry in live bird markets [1]. Closure of the markets halted new human infections, but upon reopening, several additional outbreaks occurred; 779 human infections have been documented to date according to the WHO [2]. While there are reports of possible human-to-human transmission [3–5], H7N9 has not acquired the capability for sustained transmission in the human population.

Receptor specificity of influenza A viruses is widely considered to be a barrier for transmission of avian influenza viruses in humans [6]. Over the past 50 years, the strains circulating in the human population include the H3N2 strain that caused the 1968 pandemic, a seasonal H1N1 strain introduced in 1977, and an H1N1 pandemic strain that emerged in 2009 and replaced seasonal H1N1 viruses. All human pandemic strains to date have exhibited specificity for human-type receptors (α2–6 linked), in contrast to their avian virus progenitors that recognize avian-type receptors (α2–3 linked) [7, 8]. In each case, the change in receptor specificity from avian-type to human-type involved two mutations in the HA receptor binding pocket, E190D and G225D for the H1N1 viruses, and Q226L and G228S for the H2N2 and H3N2 viruses [9, 10].

These insights have framed current efforts to determine how avian influenza with other HA serotypes might acquire human-type receptor specificity. For the H5N1 HA, introduction of the two H1 specificity-switching mutations abolished receptor binding altogether, while the H3 mutations retained avian-type receptor binding, with minimal effect on receptor specificity [11–15]. However, introducing the Q226L mutation in combination with other mutations both increased binding to human receptors and conferred respiratory droplet transmission in ferrets [16–18]. While the H7N9 virus with the Q226L mutation maintained receptor specificity for avian-type receptors, some increase in avidity for human-type receptor analogs was noted [19–21]. We therefore reasoned that additional mutations might enable a full switch to human-type receptor specificity [22].

## Results

### Mutational analyses

**Fig 1:**
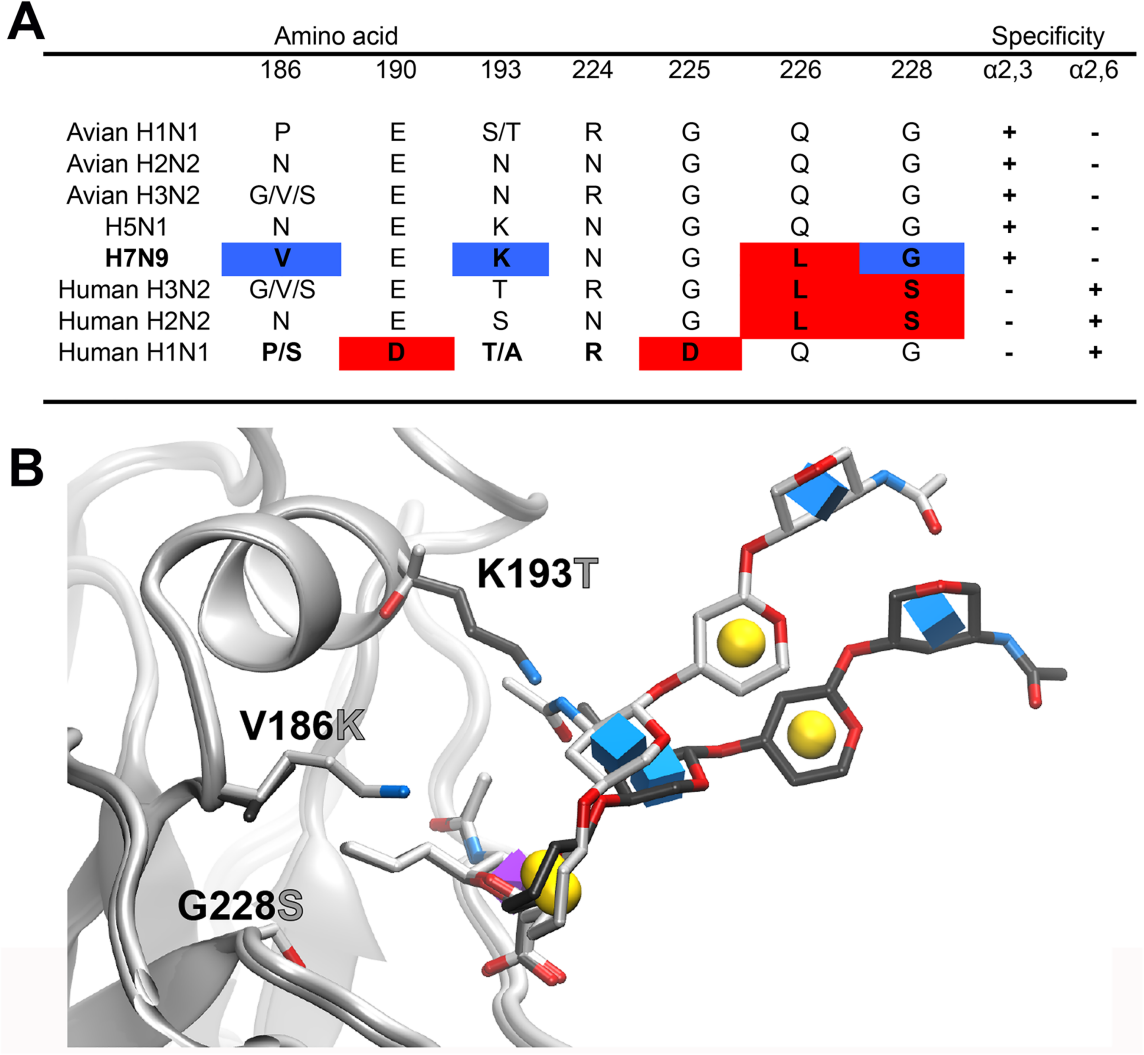
Amino acid variation in the receptor binding pocket of influenza HAs and impact of K193T mutation on receptor conformation. (A) Variation at HA positions that are known to mediate the switch in receptor binding specificity for human H1, H2 and H3 pandemic viruses and corresponding avian viruses of H1, H2, H3 and H5 subtypes in comparison with human H7N9. Red indicates amino acids involved in either human- or avian-type receptor specificity, blue indicates amino-acid positions that are mutated to the amino acids found in human H3N2 and H2N2 viruses. (B) Projection of the receptor glycan from the binding pocket. The receptor analog 6’SLNLN (α2–6 linked sialylated di-LacNAc; NeuAcα2-6Galβ1-4GlcNAcβ1-3Galβ1-4GlcNAc) is modeled in the WT H7 with K193 (dark gray), and the mutant H7 with V186K K193T G228S (light gray). In the WT, K193 causes the receptor to project further away from the 190 helix. Symbols in the sugar rings are the conventions for the Symbol Nomenclature For Glycans (SNFG) where sialic acid is the purple cubic diamond, galactose is the yellow sphere and GlcNAc is the blue cube.

We undertook a systematic mutation analysis of conserved residues in the H7 receptor-binding pocket. In addition to assessing the residues that conferred a receptor switch in the H1 and H3 hemagglutinins (Fig 1A), we focused on three other residues that might impact binding of human-type receptors. 1) In the crystal structure of H7 HA, we noted that the positively charged side chain of K193 points directly into the binding pocket [20]. This could potentially inhibit binding of extended α2–6 sialosides that are known to project over the face of the 190-loop [23]. This position is invariably a threonine or serine in human H2 and H3 viruses, respectively, and recently has been implicated to be important in the evolution of the H3N2 pandemic virus [24]. We also used molecular modeling to show that K193 would likely physically interfere with the portion of the receptor glycan that is projecting from the sialic acid bound to the receptor-binding domain (Fig 1B). 2) In a study of tissue tropism of H5N1 (A/Indonesia/05/05), a V186K mutation was found to confer binding to human trachea tissue sections. The G186V mutation was also noted as a potential adaptation of avian H7 to human-type receptors [25, 26] and, in the H2 HA, N186 has been documented to form a hydrogen bond network that enables human-type receptor binding [27]. 3) The N224K mutation was identified as a critical residue for aerosol transmission of an H5N1 virus [28].

### Receptor binding properties of H7N9 mutants that confer avian-to-human-type receptor specificity

**Fig 2:**
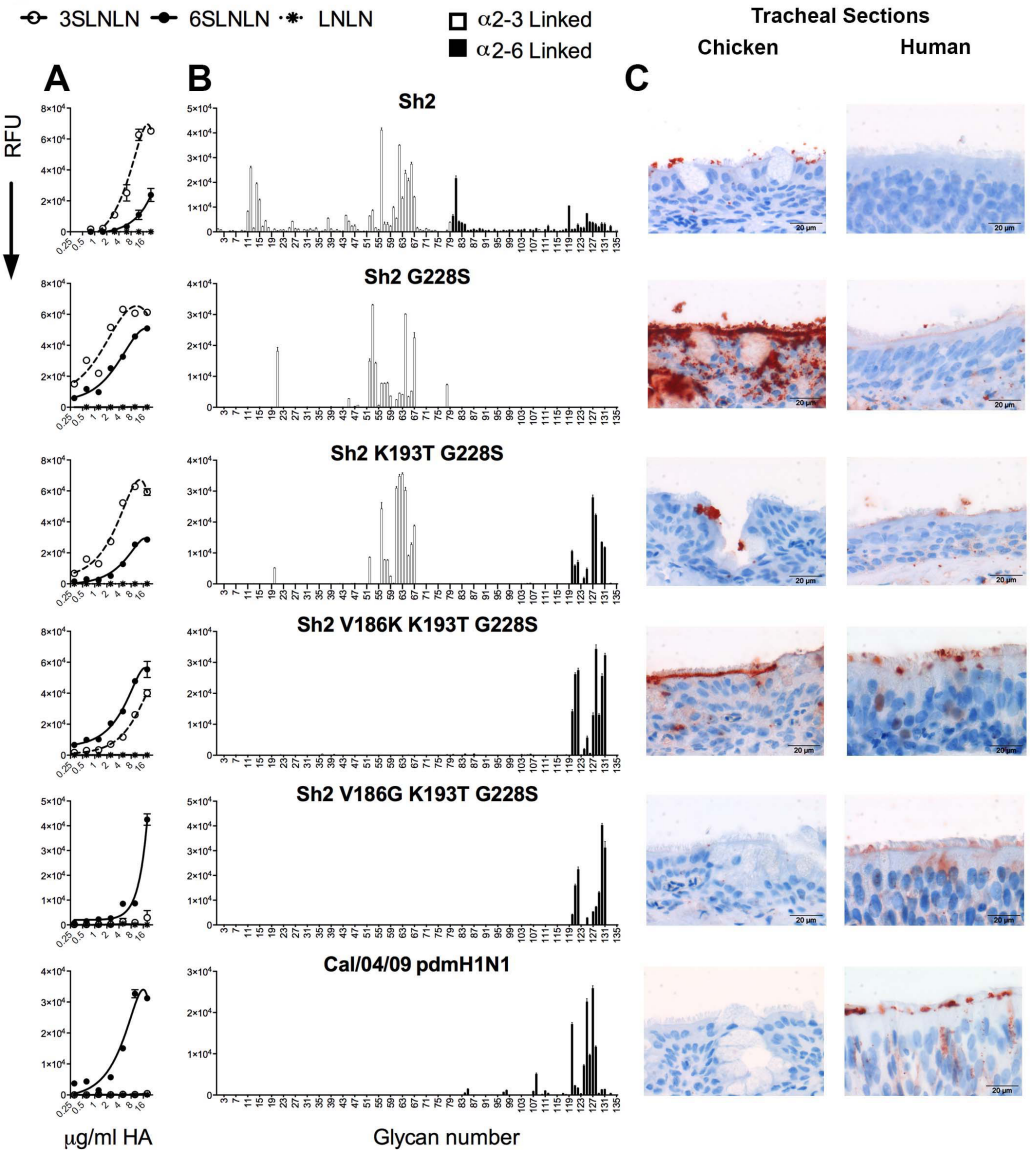
Specificity of wild type and mutant H7 HAs on glycan arrays and binding to chicken and human trachea epithelium. Glycan binding analyses of Sh2 H7N9 HA wild type and several mutants that confer human-type receptor binding: G228S, K193T G228S, V186K K193T G228S, V186G K193T G228S, with human Cal/04/09 2009 pandemic H1N1 HA as a control. (A) ELISA-like assay using sialoside polymers. The mean signal and standard error were calculated from six independent replicates; white open circles represent α2–3 linked sialylated di-LacNAc (3’SLNLN), black closed circles represent α2–6 linked sialylated di-LacNAc (6’SLNLN), and non-sialylated di-LacNAc (LNLN) are represented in asterisks. (B) The glycan array mean signal and standard error were calculated from six independent replicates; α2–3 linked sialosides are shown in white bars (glycans 11 to 79 on the x axis) and α2–6 linked sialosides in black (glycans 80 to 135). Glycans 1 to 10 are non-sialylated controls (see also S1 Table). (C) Tissue binding to either chicken or human tracheal sections is observed by HRP-staining. The sialoside array, ELISA-like assay, and tissue binding experiments are representative of three independent assays performed with different batches of HA proteins.

Varied combinations of mutations were introduced into the A/Shanghai/2/2013 (Sh2) gene and expressed as recombinant, soluble, trimeric HA proteins in HEK293S GnTI(-) cells [29]. Each recombinant HA was tested for relative avidity to α2–3 (avian-type) and α2–6 (human-type) sialoside polymers in a glycan microarray based ELISA-like assay (Fig 2A) [30, 31], and for receptor specificity using a custom glycan microarray comprising 135 sialosides with matched sets of linear receptor fragments, O-linked and N-linked glycans, each terminating in NeuAcα2-3Gal and NeuAcα2-6Gal sequences (Fig 2B, for a complete list see S1 Table) [32]. The wild-type Sh2 HA that contains the Q226L mutation has a high preference for avian-type receptors, with minimal binding to human-type receptors, as noted previously [20]. Introduction of the G228S mutation that is found in human H2 and H3 viruses retained binding to α2–3 sialosides, and gained significant but weaker binding to α2–6 sialoside in the ELISA-like assay (Fig 2A). However, there was no binding to human-type receptors in the glycan array (Fig 2B), which exhibits higher stringency [12, 16]. In contrast, mutations that confer human-type receptor specificity for H1N1 strains, E190D and G225D, alone or in combination showed no binding to sialosides in the glycan array (S3 Table).

We then introduced K193T in the G228S background. This Sh2 mutant bound almost equally well to avian-type and human-type receptors in both assays. Introduction of V186K in the K193T-G228S background, resulted in binding to human-type receptors in the ELISA-like assay, with some residual avian-type receptor binding. On the glycan array, this V186K-K193T-G228S mutant only bound human-type receptors, and displayed strikingly high specificity for α2–6 linked sialic acid found on extended N-linked glycans with 3 to 5 LacNAc repeats. A similar binding profile was also observed for an otherwise identical mutant containing V186G. The binding profile of these triple mutants is practically identical to pandemic H1N1 Cal/04/09 (Fig 2B, bottom) [32], which is known to transmit efficiently between humans.

Since most human infections to date have resulted from exposure to infected chickens in live poultry markets, we next investigated the impact of the receptor switch on binding to human and chicken airway tissues. Sh2 bound exclusively to chicken and not human trachea (Fig 2C). The G228S mutant showed very strong binding to the chicken respiratory tract and very weak yet observable binding to human trachea at the base of the cilia. For the Sh2 K193T-G228S double mutant, we observed binding to goblet cells in chicken trachea and to the base of the cilia in human trachea, consistent with this mutant having dual receptor specificity. The V186K-K193T-G228S mutant also showed dual receptor binding on chicken and human trachea sections. A triple mutant changing only V186K to V186G retained human-type receptor specificity, but exhibited reduced avidity and increased specificity for binding to human trachea epithelium, which correspond to properties similar to those of Cal/04/09 pdmH1N1.

### H7 is able to acquire human-type receptor specificity

**Fig 3:**
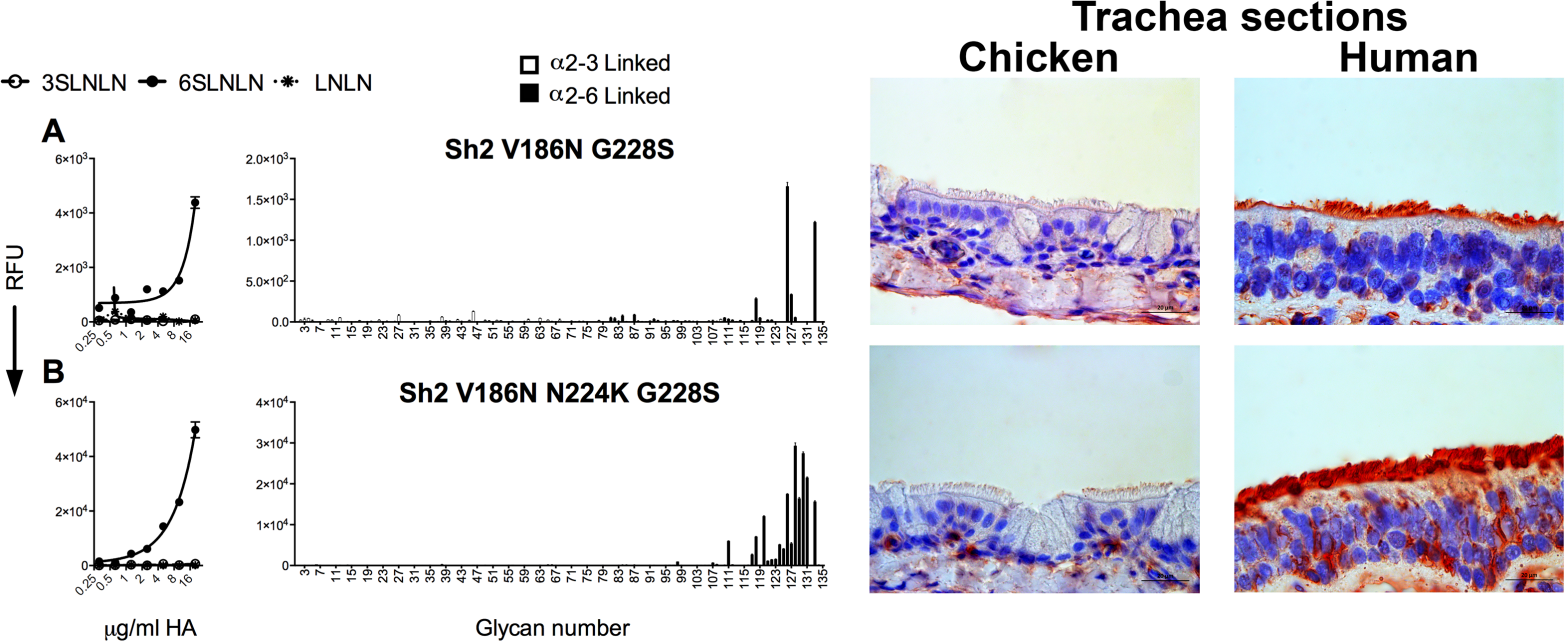
H7 Sh2 mutant combinations that also bind to human-type receptors. Glycan binding analyses of Sh2 H7N9 mutant HAs, V186N G226S (A) and V186N N224K G228S (B). The mean signal and standard error were calculated from six independent replicates on both the PAA (left column) and the sialoside array (right column). Tissue binding to either chicken or human tracheal sections is observed by HRP-staining (right column). In the PAA array, white open circles represent α2–3 linked sialylated di-LacNAc (3’SLNLN), black closed circles represent α2–6 linked sialylated di-LacNAc (6’SLNLN), and non-sialylated di-LacNAc (LNLN) are represented in asterisks. In the sialoside array α2–3 linked sialosides are shown in white bars (glycans 11 to 79 on the x axis) and α2–6 linked sialosides in black (glycans 80 to 135). Glycans 1 to 10 are non-sialylated controls (see also S1 Table). The sialoside array, ELISA-like assay and tissue binding experiments shown are representative of three independent assays performed with different batches of HA proteins.

On analysis of the V186N and N224K mutations, we found that V186N in just the G228S background led to specific binding to human-type receptors in both the ELISA-like assay with sialoside polymers (Fig 3A) and the glycan array (Fig 3B). With the addition of N224K in the V186N-G228S background, we observed a significant increase in binding. Thus, there are multiple ways for H7N9 to obtain human-type receptor specificity. The N224K mutant does not confer specificity to human-type receptors in other Sh2 backgrounds (S3 Table), but does increase binding in human-specific Sh2 mutants (S1 Fig). We conclude that a lysine at position 224 does not significantly alter receptor specificity, but does enhance the strength of binding, likely through a positive avidity contribution [28].

#### Binding avidity of H7 HAs to N-linked glycans

**Fig 4:**
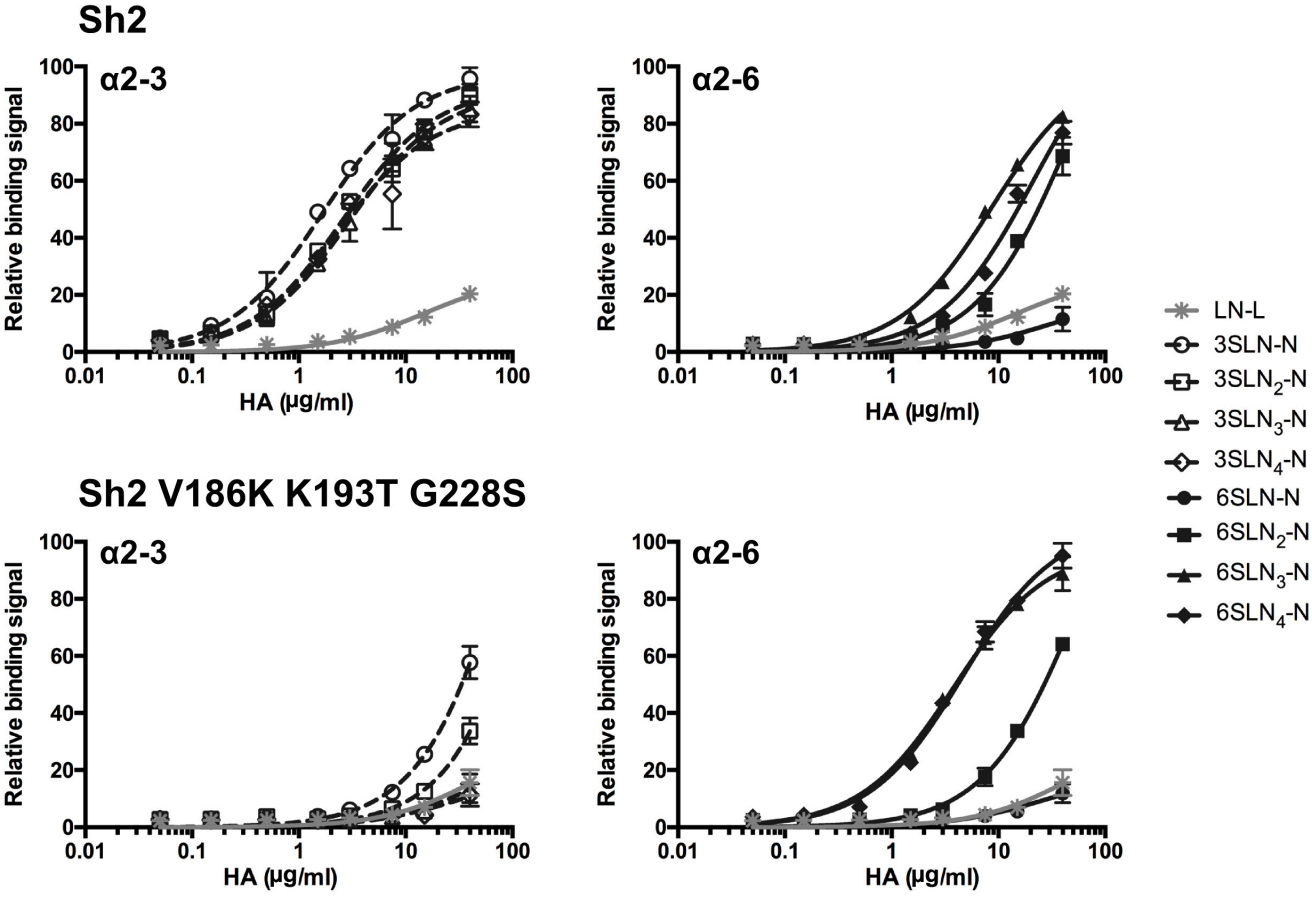
Avidity of Sh2 (WT) and Sh2 V186K-K193T-G228S variant HA for N-linked glycan receptors assessed by glycan ELISA. Sh2 (upper panels) binds strongly to avian-type (α2–3) receptors (left, white open shapes) with weaker binding to human-type (α2–6) receptors (right, black closed shapes). Sh2 V186K-K193T-G228S (lower panels) shows vastly reduced avidity for avian N-glycans and increased selectivity for extended glycan receptors to human receptors. Assays are conducted with biantennary, N-linked glycans (N) with one to four LacNAc (LN, Galβ1-4GlcNAc) repeats terminated with sialic acid (S) in α2–3 or α2–6 linkage (SLN_1-4_-N). An asialo, mono-LacNAc (LacNAc-biotin, LN-L) was used as a negative binding control.

To quantify the binding avidities of H7 Sh2 and mutant (V186K-K193T-G228S) HAs, and assess in detail the strength of the specificity switch to human-type receptor binding, we conducted a glycan ELISA using a series of biantennary N-linked glycans featuring either terminal NeuAcα2-3Gal or NeuAcα2-6Gal. These glycans are consistently observed as preferred receptors on the glycan array [32]. Sh2 was selective for avian-type receptors, with weaker binding to human-type receptors, and showed little preference for glycan length, consistent with the glycan array results (Fig 4 top). The Sh2 V186K-K193T-G228S mutant lost almost all binding to avian-type receptors, and binds with higher avidity to human-type receptors than the wild-type Sh2 (Fig 4 bottom). Interestingly, the increased avidity of the mutant was seen primarily for extended α2-6-linked N-glycans, with 3 or 4 LacNAc repeats, with a 2- to 5-fold gain (apparent *K*_d_ values are given in S4 Table). These data are consistent with our hypothesis that HAs with human-type receptor specificity accommodate not only the altered chemistry of the terminal sialoside linkage, but also permit bidentate binding, resulting in an apparent preference for biantennary N-glycans with 3 or more LacNAc repeats [32].

#### Thermostability of H7 HAs

**Fig 5:**
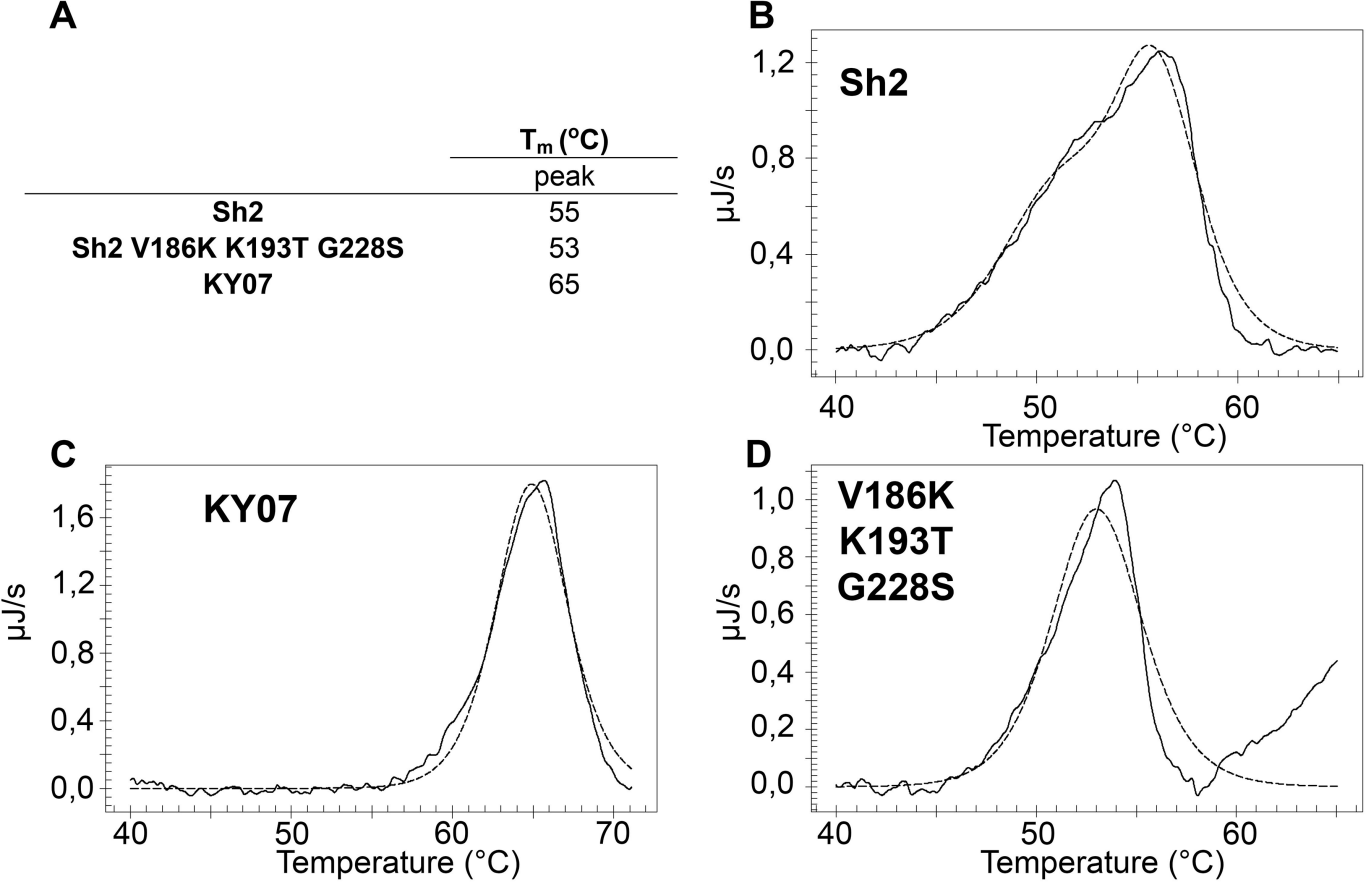
Melting curve of recombinant HA obtained by DSC to determine the thermostability of Sh2, Sh2 V186K K193T G228S, and A/KY/07. The raw data are depicted in the solid line, while the fitted curve, from which the *T*_m_ was derived, is depicted with a dotted line. (A) A summary of the peaks observed during the DSC experiments for each recombinant HA, (B) Sh2, (C) A/KY/07 and (D) Sh2 V186K K193T G228S.

The stability of HA in acidic environments is a determinant for airborne transmissibility of influenza viruses among humans [33]. In general, human virus HAs exhibit increased stability and fuse at lower pH than avian virus HAs. Thermal stability of an HA correlates with stability to fuse at low pH, and can be used as an alternative measure of HA stability [33, 34]. Using differential scanning calorimetry (DSC), we analyzed the thermostability of Sh2 and Sh2 V186K-K193T-G228S proteins relative to a human seasonal H1N1 control, A/KY/07. Sh2 exhibited a broad thermal denaturation profile with a melting temperature (*T*_m_) of 55°C, while the human-type receptor specificity mutant (Sh2 V186K-K193T-G228S) had a slightly lower stability with a *T*_*m*_ of 53°C (Fig 5). While H7N9 has been demonstrated to transmit between ferrets at low efficiency [35, 36], the lower stability of the mutant would suggest it is less likely to transmit than the wild type. In contrast to the H7 HAs, the H1 HA of A/KY/07 human control had a *T*_m_ of 65°C, indicative of the higher stability expected of a viral HA that transmits in humans. Thus, while the Sh2 V186K-K193T-G228S mutant exhibits human-type receptor specificity, the thermal stability is, if anything, lower than wild-type Sh2 HA, which is not surprising since mutations that increase stability are generally in the stem region, not in the receptor binding domain.

### K193T permits bidendate receptor-binding to N-linked glycans

**Fig 6:**
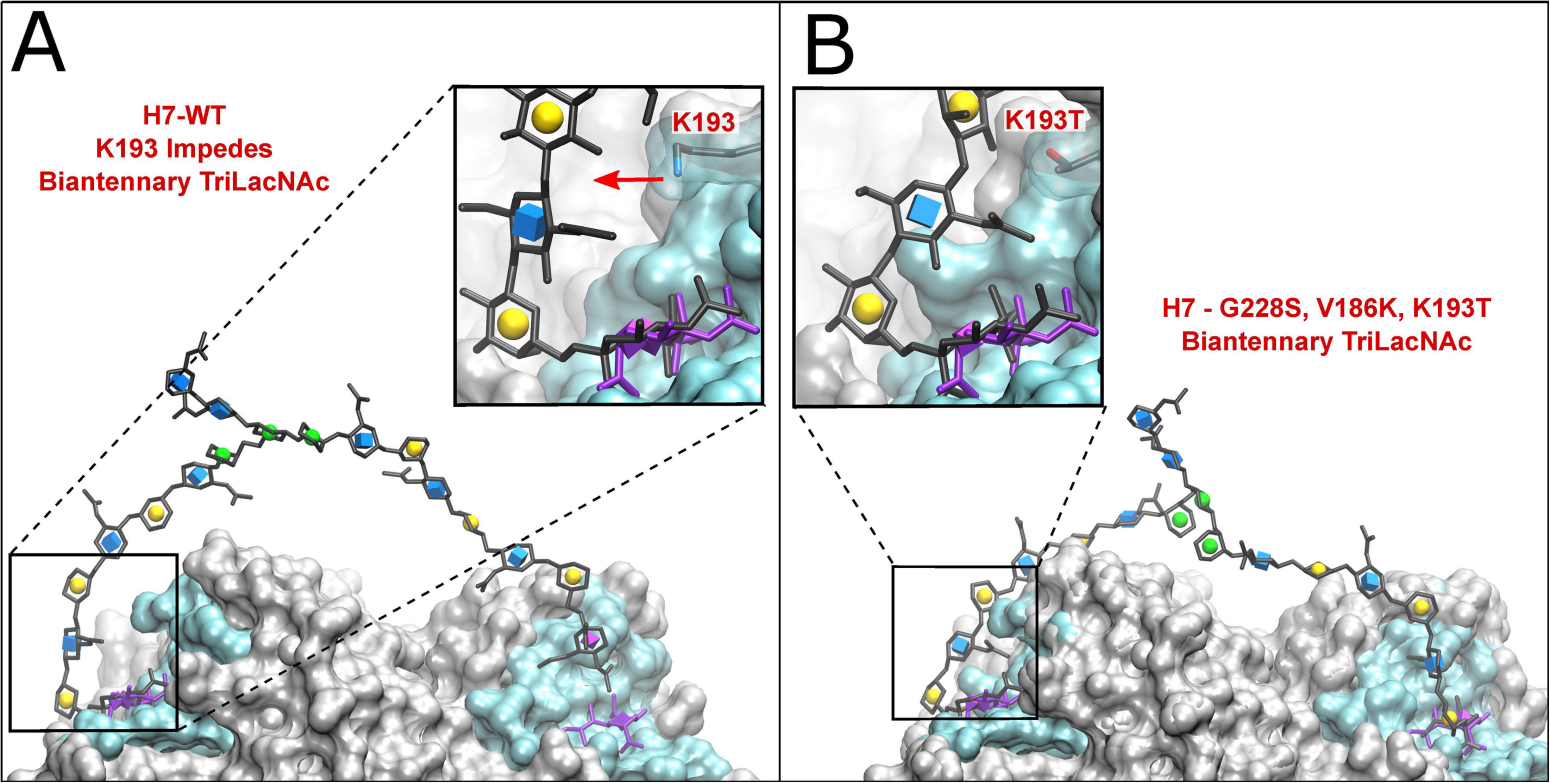
Modeling of bidendate binding for biantennary tri-LacNAc N-glycans to WT and V186K, K193T G228S triple mutant (TM) H7. The 6’SLN_3_-N glycan was impeded by K193 in the WT (A), but was able to span two binding sites in the TM H7 (B).

The influence of the K193T mutation on human-type receptor binding was of particular interest because K193S was shown to be an essential mutation for the H3 Hong-Kong 1968 pandemic, and K193T for H10N8 to obtain binding to human-type receptors [24, 37]. In an ideal *cis* conformation, the human-type receptor would bind and project from the sialic acid binding site towards the 190 helix and has the potential to interact with amino acids of the 190 helix that frame the top of the receptor binding site [23]. Moreover, we have recently shown that H3N2 viruses, as well as pandemic H1N1, exhibit preference for branched N-linked glycans that feature elongated LacNAc repeats extending over the 190 helix. These receptors project the second branch over the top of HA such that the second sialic acid can reach the receptor binding site of a second protomer in the same trimer [32]. Using molecular modeling, we investigated the possibility that the K193T mutation in Sh2 H7N9 HA would impact simultaneous binding of human-type receptors on complex N-glycans, as shown in Fig 6. Here the low-energy conformation of the extended glycan chain produces a steric clash with the K193, forcing LacNAc moieties to adopt a conformation projecting out of the receptor-binding site, and away from the 190 helix. Such a clash likely disfavors the preferred binding mode, where the rest of the glycan arches over the top of the HA surface. As a result, bidendate binding involving the simultaneous coordination of another branch of the glycan to a second protomer in the HA trimer is not possible (Fig 6A). In contrast, simulations show that T193 interacts with LacNAc, enabling it to come closer to the HA, facilitating a bidentate interaction where the glycan is able to extend over the top of the trimer, thus effectively increasing avidity (Fig 6B).

We also determined the crystal structure of the Sh2 V186K K193T G228S with and without avian- and human-type receptors (S2A and S2B Fig). The structures were virtually identical compared to the previously determined crystal structure of the Sh2 H7 HA protein (Protein Data Bank [PDB] code 4N5J [20]). Moreover, in co-crystals with monomeric human-type (LSTc) and avian-type (LSTa) receptor analogs, electron density is seen only for the sialic acid, consistent with low-affinity binding of the monovalent receptor to the receptor site (S3 Fig) and preference of the mutants for extended biantennary glycans that offer the potential for bidentate binding.

## Discussion

We demonstrate here that several alternative three-amino-acid mutations (V186G/K-K193T-G228S or V186N-N224K-G228S) can switch the receptor specificity of the H7N9 HA from avian- to human-type, a property required for transmission in humans and ferrets [38, 39]. Of these mutations, only isolated examples of 186G and 193N have to date been reported in H7 avian isolates. The mutants show profound loss of binding to avian-type (α2–3 linked) receptors, and increased binding to human-type (α2–6 linked) receptors in both glycan microarrays and glycan ELISA-type avidity assays. The mutants exhibit preferential binding to a subset of human-type receptors with extended branched N-linked glycans that terminate with NeuAcα2-6Gal reported to be present in N-linked glycans in human and ferret airway tissues [23, 40, 41]. Notably, this specificity for a restricted subset of human-type receptors is shared with recent H3N2 viruses, and the 2009 H1N1 pandemic virus. We have also recently observed that different sets of mutations switch the H6N1 and H10N8 HAs to human-type receptor specificity and, in each case, confer specificity for a similar subset of human-type receptors [37] (de Vries, Tzarum, Wilson & Paulson manuscript in revision). Thus, recognition of human-type receptors with extended glycan chains appears to be a common characteristic of human influenza virus HAs, and avian virus HA mutants that bind to human-type receptors.

Ideally, it would be important to assess the impact of the switch in receptor specificity in the ferret model that displays human-type receptors in the airway epithelium and is used to assess the propensity for air droplet transmission of human viruses. However, the introduction of the mutations that switch receptor specificity into an actual H7N9 virus background would represent gain-of-function (GoF) experiments that are currently prohibited [42]. During the course of this study, no viruses were created, and no experiments assessing the potential for air droplet transmission were performed. We suggest that understanding mutations that can confer human-type receptor binding will benefit risk assessment in worldwide surveillance of H7N9 in poultry and humans.

## Materials and methods

### Ethics statement

IRB & IACUC & IBC approval obtained at the funded institution. The tissues used for this study were obtained from the tissue archive of the Veterinary Pathologic Diagnostic Center (Department of Pathobiology, Faculty of Veterinary Medicine, Utrecht University, The Netherlands). This archive is composed of paraffin blocks with tissues maintained for diagnostic purposes; no permission of the Committee on the Ethics of Animal Experiment is required. Anonymized human tissues were obtained under Service Level Agreement from the University Medical Centre Utrecht, The Netherlands. Use of anonymous material for scientific purposes is part of the standard treatment contract with patients and therefore informed consent procedure was not required according to the institutional medical ethical review board."

### Expression and purification of HA for binding studies

Codon-optimized H1 and H7 encoding cDNAs (Genscript, USA) of A/Shanghai/2/13, Cal/04/09 and A/KY/07 were cloned into the pCD5 expression as described previously [29]. The pCD5 expression vector is adapted so that that the HA-encoding cDNAs are cloned in frame with DNA sequences coding for a signal sequence, a GCN4 trimerization motif (RMKQIEDKIEEIESKQKKIENEIARIKK), and Strep-tag II (WSHPQFEK; IBA, Germany).

The HA proteins were expressed in HEK293S GnTI(-) cells (ATCC) and purified from the cell culture supernatants as described previously [43]. pCD5 expression vectors were transfected into HEK293S GnTI(-) cells using polyethyleneimine I (PEI). At 6 h post transfection, the transfection mixture was replaced by 293 SFM II expression medium (Gibco), supplemented with sodium bicarbonate (3.7 g/liter), glucose (2.0 g/liter), Primatone RL-UF (Kerry) (3.0 g/liter), penicillin (100 units/ml), Streptomycin (100 μg/ml), glutaMAX (Gibco), and 1.5% DMSO. Tissue culture supernatants were harvested 5–6 days post transfection. HA proteins were purified using Strep-Tactin sepharose beads according to the manufacturer’s instructions (IBA, Germany)

### Glycan microarray binding of HA

Purified, soluble trimeric HA was pre-complexed with horseradish peroxidase (HRP)-linked anti-Strep-tag mouse antibody (IBA) and with Alexa488-linked anti-mouse IgG (4:2:1 molar ratio) prior to incubation for 15 min on ice in 100 μl PBS-T, and incubated on the array surface in a humidified chamber for 90 minutes. Slides were subsequently washed by successive rinses with PBS-T, PBS, and deionized H_2_O. Washed arrays were dried by centrifugation and immediately scanned for FITC signal on a Perkin-Elmer ProScanArray Express confocal microarray scanner. Fluorescent signal intensity was measured using Imagene (Biodiscovery) and mean intensity minus mean background was calculated and graphed using MS Excel. For each glycan, the mean signal intensity was calculated from 6 replicate spots. The highest and lowest signals of the 6 replicates were removed and the remaining 4 replicates used to calculate the mean signal, standard deviation (SD), and standard error measurement (SEM). Bar graphs represent the averaged mean signal minus background for each glycan sample and error bars are the SEM value. A list of glycans on the microarray is included in S1 Table.

### Glycan ELISA

Purified HA trimers were precomplexed with anti-HIS mouse IgG (Invitrogen) and HRP-conjugated goat anti-mouse IgG (Pierce), then diluted in series to required assay concentrations (40–0.05 μg/mL final). Preparation of streptavidin-coated plates with biotinylated glycans, incubation and washing of pre-complexed HA dilutions was exactly as described previously [23, 32].

### Tissue staining

Sections of formalin-fixed, paraffin-embedded, human trachea and chicken trachea were obtained from the University Medical Center and the Department of Veterinary Pathobiology, Faculty of Veterinary Medicine, at Utrecht University, respectively. Tissue sections were rehydrated in a series of alcohol from 100%, 96% and 70%, and lastly in distilled water. Endogenous peroxidase activity was blocked with 1% hydrogen peroxide for 30 min at room temperature. Tissue slides were boiled in citrate buffer pH 6.0 for 10 minutes at 900kW in a microwave for antigen retrieval and washed in PBS-T three times. Tissue was subsequently incubated with 3% BSA in PBS-T for overnight at 4^°^C. On the next day, the purified HAs were precomplexed with mouse anti-strep-tag- HRP antibodies (IBA) and goat anti-mouse IgG HRP antibodies (Life Biosciences) at a 4:2:1 ratio in PBS-T with 3% BSA and incubated on ice for 20 minutes. After draining the slide, the precomplexed HA was applied onto the tissue and incubated for 90 minutes at RT. Sections were then washed in PBS-T, incubated with 3-amino-9-ethyl-carbazole (AEC; Sigma-Aldrich) for 15 minutes, counterstained with hematoxylin, and mounted with Aquatex (Merck). Images were taken using a charge-coupled device (CCD) camera and an Olympus BX41 microscope linked to CellB imaging software (Soft Imaging Solutions GmbH, Münster, Germany).

### Cloning, baculovirus expression and purification of ha for crystallization

The ectodomain of the Sh2 H7 HA mutant (V186K-K193T-G228S) was expressed in a baculovirus system essentially as previously described [20]. Briefly, the cDNAs corresponding to residues 19–327 of HA1 and 1–174 of HA2 (H3 numbering) of HA from A/Shanghai/2/2013 (H7N9) (Global Initiative on Sharing All Influenza Data (GISAID) isolate ID: EPI_ISL_138738) were codon-optimized and synthesized for insect cell expression and inserted into a baculovirus transfer vector, pFastbacHT-A (Invitrogen) with an N-terminal gp67 signal peptide, C-terminal trimerization domain, His_6_ tag, and thrombin cleavage site incorporated to separate the HA ectodomain and the trimerization and His tags. The HA1 domain triple mutant (G228S, V186K, K193T) was made by site-directed mutagenesis. The purified recombinant HA Bacmids were used to transfect Sf9 insect cells for overexpression. HA protein was produced in infecting suspension cultures of Hi5 cells with recombinant baculovirus at an MOI of 5–10 and incubated at 28°C shaking at 110 RPM. After 72 hours, Hi5 cells were removed by centrifugation and supernatants containing secreted, soluble HA proteins were concentrated and buffer-exchanged into 1xPBS, pH 7.4. The HAs were recovered from the cell supernatants by metal affinity chromatography using Ni-NTA resin, and were digested with thrombin to remove the trimerization domain and His_6-_tag. The cleaved HAs were further purified by size exclusion chromatography on a Hiload 16/90 Superdex 200 column (GE healthcare, Pittsburgh, PA) in 20 mM Tris pH 8.0, 100 mM NaCl, and 0.02% (v/v) NaN_3_.

### 3D structure generation

Structure models were generated from PDBID 4LN8 [44]. A trimeric “head region” was created from residues K46 to S260 from the HA1 (receptor binding region) and residues Q61 to S93 from HA2 (from the top of membrane fusion stem region). One structure was kept as WT, while each of the three binding sites in a second structure were altered by point mutations; G228S, V186K and K193T. The mutant model structures were generated in UCSF Chimera [45], by selecting rotamers from the Dunbrack library [46]. A sialylated biantennary TriLacNAc N-glycan was generated on Glycam-Web (www.glycam.org/cb) and modeled into both the WT and mutated structure via computational carbohydrate grafting [47], using the Neu5Ac in PDBID 4LN8 as a template. The reported grafting algorithm [48, 49] was adapted to rotate the glycosidic linkages within normal bounds [50], while monitoring the distance between the binding motif on the other arm of the glycan and the second HA binding site. The linkages were adjusted in series, beginning from the NeuAcα2-6Gal motif. A single optimal structure was selected based on the relative orientation and proximity of the NeuAcα2-6Gal motif on the other arm to the target HA binding site. The results were independent of whether the 3-arm or the 6-arm of the glycan was grafted onto the bound NeuAcα2-6Gal motif. The resulting structures were then subject to energy minimization and molecular dynamics simulation as described previously, to attempt to see whether the NeuAcα2-6Gal motif on the second arm of the glycan could locate into second binding site.

### Crystallization, data collection and structural determination

Crystallization experiments were set up using the sitting drop vapor diffusion method. Initial crystallization conditions for the H7 mutant HA (V186K-K193T-G228S) were obtained from robotic crystallization trials using the automated CrystalMation system (Rigaku) at The Scripps Research Institute. Following optimization, diffraction quality crystals of the triple mutant HA were grown at 22°C by mixing 0.5 μl of protein (7.4 mg/ml) in 20 mM Tris, pH 8.0, 100 mM NaCl with 0.5 μl of a reservoir solution containing 0.2 M tri-potassium citrate, 5% (v/v) ethylene glycol and 22% (w/v) PEG3350. The crystals were flash-cooled in liquid nitrogen by adding 20% (v/v) ethylene glycol to the mother liquor as cryoprotectant. The triple mutant HA-ligand complexes were obtained by soaking HA crystals in the well solution that now contained glycan ligands. Final concentrations of ligands LSTa (NeuAcα2-3Galβ1-3GlcNAcβ1-3Galβ1-4Glc) and LSTc (NeuAcα2-6Galβ1-4GlcNAcβ1-3Galβ1-4Glc) were all 5 mM, and soaking times were 10 min. Diffraction data were collected on synchrotron radiation sources specified in the data statistics tables. HKL2000 (HKL Research, Inc.) was used to integrate and scale diffraction data. Initial phases were determined by molecular replacement using Phaser [51] with the wild-type HA structure (PDB codes 4N5J) as a model. One HA protomer is present per asymmetric unit. Refinement was carried out using the program Phenix [52]. Model rebuilding was performed manually using the graphics program Coot [53]. Final refinement statistics are summarized in S2 Table.

### Differential scanning calorimetry (DSC)

Thermal denaturation was studied using a nano-DSC calorimeter (TA instruments, Etten-Leur, The Netherlands). HA proteins were eluted from the streptavidin beads in PBS with 2.5mM desthiobiotin, and 100μg of protein was tested. After loading the sample into the cell, thermal denaturation was probed at a scan rate of 60°C/h. Buffer correction, normalization, and baseline subtraction procedures were applied before the data were analyzed using NanoAnalyze Software v.3.3.0 (TA Instruments). The data were fitted using a non-two-state model.

### Accession numbers

Atomic coordinates and structure factors have been deposited in the Protein Data Bank (PDB) under accession codes 5VJK, 5VJL and 5VJM for Sh2 mutant HA (V186K-K193T-G228S) in apo form and in complex with LSTc or LSTa.

## Supporting information

S1 Table Glycans imprinted on the sialoside array. (PDF)

S2 Table Data collection and refinement statistics for Sh2 H7 triple mutant (V186K, K193T, G228S). (PDF)

S3 Table Receptor binding of H7 mutants. (PDF)

S4 Table Kd affinities for Sh2 and Sh2 V186K K193T G228S to biantennary N-glycans with either human- or avian-type receptors on different numbers of LacNAc repeats. (PDF)

S1 Fig Receptor binding of SH2 N224K mutants. (PDF)

S2 Fig Crystal structures of human Sh2 H7N9 HA mutants. (PDF)

S3 Fig Simulated annealing omit (Fo-Fc) electron density maps of glycan ligands bound to the H7 HA triple mutant. (PDF)
