# Supplementary material for: Three mutations switch H7N9 influenza to human-type receptor specificity
Source: PLoS Pathog. 2017 Jun 15;13(6):e1006390. doi: 10.1371/journal.ppat.1006390 (PMC5472306; doi:10.1371/journal.ppat.1006390)
Supplement: S1 Table — (PDF) [file ppat.1006390.s001.pdf]

S1 Table. Glycans imprinted on the sialoside array with non-sialylated controls #1-10 in gray, avian-type receptors #11-79 in white and human-type receptors #80-135 in black. Purple diamonds represent sialic acid, yellow circles represent Galactose, blue circles represent Glucose, green circles represent Mannose, yellow squares represent N-Acetyl-Galactosamine, blue squares represent N-Acetyl-Glucosamine and red triangles represent Fucose.

Glycan #	Common Name	Structure
1	Galβ(1-4)GlcNAcβ-ethyl-NH2	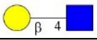
2	Galβ(1-4)GlcNAcβ(1-3)Galβ(1-3)GalNAcα-Thr-NH2	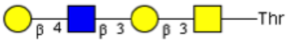
3	Galβ(1-4)GlcNAcβ(1-6)[Galβ(1-3)]-GalNAcα-Thr-NH2	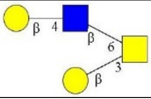
4	Galβ(1-4)GlcNAcβ(1-3)GalNAcα-Thr-NH2	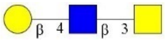
5	Galβ(1-4)GlcNAcβ(1-3)[Galβ(1-4)GlcNAcβ(1-6)]-GalNAcα-Thr-NH2	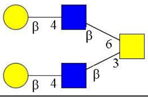
6	Galβ(1-4)GlcNAcβ(1-6)GalNAcα-Thr-NH2	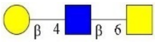
7	Galβ(1-4)GlcNAcβ(1-2)Manα (1-3)[Galβ(1-4)GlcNAcβ(1-2)Manα(1-6)]-Manβ(1-4)GlcNAcβ(1-4)GlcNAcβ-Asn-NH2	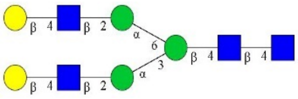
8	Galβ(1-4)GlcNAcβ(1-2)Manα(1-3)[Galβ(1-4)GlcNAcβ(1-2)Manα(1-6)]-Manβ(1-4)GlcNAcβ(1-4)[Fucα(1-6)]-GlcNAcβ-Asn-Ser-Thr-NH2	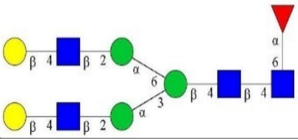
9	Galβ(1-4)GlcNAcβ(1-2)Manα(1-3){Galβ(1-4)GlcNAcβ(1-2)[Galβ(1-4)GlcNAcβ(1-2)]-Manα(1-6)}-Manβ(1-4)GlcNAcβ(1-4)GlcNAcβ-Asn-Lys-NH2	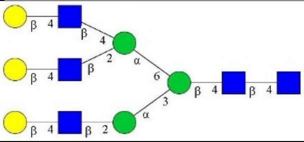
10	Galβ(1-4)GlcNAcβ(1-2)Manα(1-3){Galβ(1-4)GlcNAcβ(1-2)[Galβ(1-4)GlcNAcβ(1-2)]-Manα(1-6)}-Manβ(1-4)GlcNAcβ(1-4)[Fucα(1-6)]-GlcNAcβ-(Lys-Val-Ala)Asn-Lys-Thr-NH2	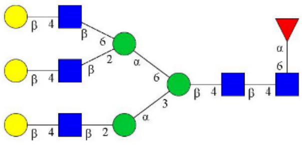
11	NeuAcα(2-3)Galβ(1-4)6-O-sulfo-GlcNAcβ-propyl-NH2	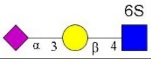
12	NeuAcα(2-3)Galβ(1-4)[Fucα(1-3)]-6-O-sulfo-GlcNAcβ-propyl-NH2	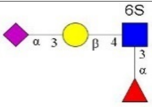
13	NeuAcα(2-3)6-O-sulfo-Galβ(1-4)GlcNAcβ-ethyl-NH2	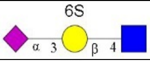
14	NeuAcα(2-3)6-O-sulfo-Galβ(1-4)[Fucα(1-3)]-GlcNAcβ-propyl-NH2	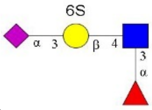
15	NeuAcα(2-3)Galβ(1-3)6-O-sulfo-GlcNAcβ-propyl-NH2	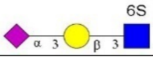
16	NeuAcα(2-3)Galβ(1-4)Glcβ-ethyl-NH2	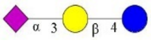

Glycan #	Common Name	Structure
17	NeuAcα(2-3)Galβ(1-4)GlcNAcβ-ethyl-NH2	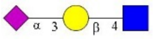
18	NeuAcα(2-3)Galβ(1-4)GlcNAcβ(1-3)Galβ(1-4)GlcNAcβ-ethyl-NH2	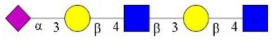
19	NeuAcα(2-3)Galβ(1-4)GlcNAcβ(1-3)Galβ(1-4)GlcNAcβ(1-3)Galβ(1-4)GlcNAcβ-ethyl-NH2	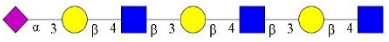
20	NeuAcα(2-3)GalNAcβ(1-4)GlcNAcβ-ethyl-NH2	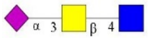
21	NeuAcα(2-3)Galβ(1-3)GlcNAcβ-ethyl-NH2	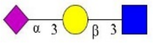
22	NeuAcα(2-3)Galβ(1-3)GlcNAcβ(1-3)Galβ(1-4)GlcNAcβ-ethyl-NH2	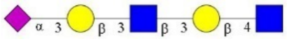
23	NeuAcα(2-3)Galβ(1-3)GlcNAcβ(1-3)Galβ(1-3)GlcNAcβ-ethyl-NH2	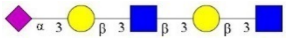
24	NeuAcα(2-3)Galβ(1-3)GalNAcβ(1-3)Galα(1-4)Galβ(1-4)Glcβ-ethyl-NH2	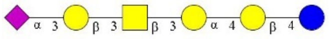
25	NeuAcα(2-3)Galβ(1-3)GalNAcα-Thr-NH2	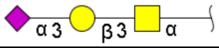
26	NeuAcα(2-3)Galβ(1-4)GlcNAcβ(1-3)Galβ(1-3)GalNAcα-Thr-NH2	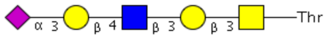
27	NeuAcα(2-3)Galβ(1-4)GlcNAcβ(1-3)Galβ(1-4)GlcNAcβ(1-3)Galβ(1-3)GalNAcα-Thr-NH2	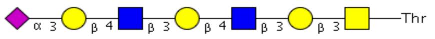
28	NeuAcα(2-3)Galβ(1-4)GlcNAcβ(1-3)Galβ(1-4)GlcNAcβ(1-3)Galβ(1-4)GlcNAcβ(1-3)Galβ(1-3)GalNAcα-Thr-NH2	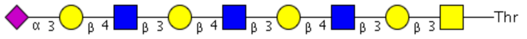
29	NeuAcα(2-3)Galβ(1-4)GlcNAcβ(1-3)Galβ(1-4)GlcNAcβ(1-3)Galβ(1-4)GlcNAcβ(1-3)Galβ(1-4)GlcNAcβ(1-3)Galβ(1-3)GalNAcα-Thr-NH2	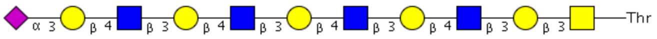
30	NeuAcα(2-3)Galβ(1-4)GlcNAcβ(1-3)Galβ(1-4)GlcNAcβ(1-3)Galβ(1-4)GlcNAcβ(1-3)Galβ(1-4)GlcNAcβ(1-3)Galβ(1-4)GlcNAcβ(1-3)Galβ(1-3)GalNAcα-Thr-NH2	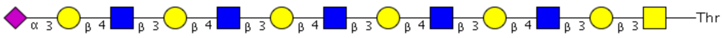
31	NeuAcα(2-3)Galβ(1-3)[GlcNAcβ(1-6)]-GalNAcα-Thr-NH2	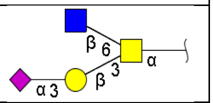
32	NeuAcα(2-3)Galβ(1-4)GlcNAcβ(1-6)[Galβ(1-3)]-GalNAcα-Thr-NH2	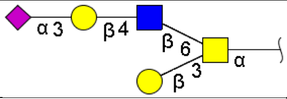
33	NeuAcα(2-3)Galβ(1-4)GlcNAcβ(1-3)Galβ(1-4)GlcNAcβ(1-6)[Galβ(1-3)]-GalNAcα-Thr-NH2	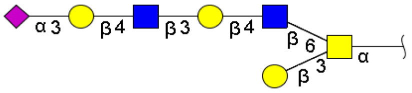
34	NeuAcα(2-3)Galβ(1-4)GlcNAcβ(1-3)Galβ(1-4)GlcNAcβ(1-3)Galβ(1-4)GlcNAcβ(1-6)[Galβ(1-3)]-GalNAcα-Thr-NH2	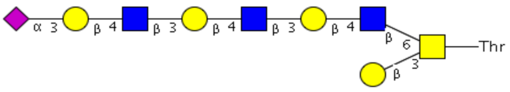
35	NeuAcα(2-3)Galβ(1-4)GlcNAcβ(1-3)Galβ(1-4)GlcNAcβ(1-3)Galβ(1-4)GlcNAcβ(1-3)Galβ(1-4)GlcNAcβ(1-6)[Galβ(1-3)]-GalNAcα-Thr-NH2	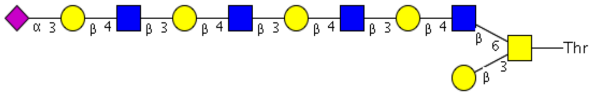
36	NeuAcα(2-3)Galβ(1-4)GlcNAcβ(1-3)Galβ(1-4)GlcNAcβ(1-3)Galβ(1-4)GlcNAcβ(1-3)Galβ(1-4)GlcNAcβ(1-3)Galβ(1-4)GlcNAcβ(1-6)[Galβ(1-3)]-GalNAcα-Thr-NH2	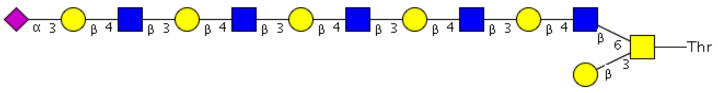

Glycan #	Common Name	Structure
37	NeuAcα(2-3)Galβ(1-4)GlcNAcβ(1-3)Galβ(1-4)GlcNAcβ(1-3)Galβ(1-4)GlcNAcβ(1-3)Galβ(1-4)GlcNAcβ(1-6)[NeuAcα(2-3)Galβ(1-4)GlcNAcβ(1-3)Galβ(1-4)GlcNAcβ(1-3)Galβ(1-4)GlcNAcβ(1-3)Galβ(1-3)]-GalNAcα-Thr-NH2	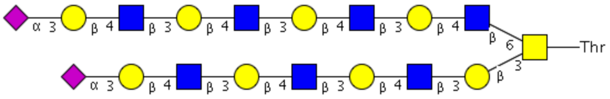
38	NeuAcα(2-3)Galβ(1-4)GlcNAcβ(1-3)Galβ(1-4)GlcNAcβ(1-3)Galβ(1-4)GlcNAcβ(1-3)Galβ(1-4)GlcNAcβ(1-3)Galβ(1-4)GlcNAcβ(1-6)[NeuAcα(2-3)Galβ(1-4)GlcNAcβ(1-3)Galβ(1-4)GlcNAcβ(1-3)Galβ(1-4)GlcNAcβ(1-3)Galβ(1-4)GlcNAcβ(1-3)Galβ(1-3)]-GalNAcα-Thr-NH2	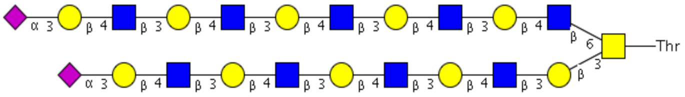
39	NeuAcα(2-3)Galβ(1-4)GlcNAcβ(1-3)GalNAcα-Thr-NH2	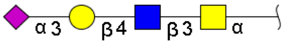
40	NeuAcα(2-3)Galβ(1-4)GlcNAcβ(1-3)Galβ(1-4)GlcNAcβ(1-3)GalNAcα-Thr-NH2	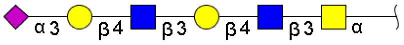
41	NeuAcα(2-3)Galβ(1-4)GlcNAcβ(1-3)Galβ(1-4)GlcNAcβ(1-3)Galβ(1-4)GlcNAcβ(1-3)GalNAcα-Thr-NH2	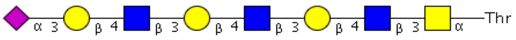
42	NeuAcα(2-3)Galβ(1-4)GlcNAcβ(1-3)Galβ(1-4)GlcNAcβ(1-3)Galβ(1-4)GlcNAcβ(1-3)Galβ(1-4)GlcNAcβ(1-3)GalNAcα-Thr-NH2	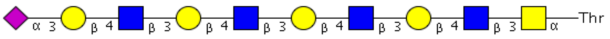
43	NeuAcα(2-3)Galβ(1-4)GlcNAcβ(1-3)Galβ(1-4)GlcNAcβ(1-3)Galβ(1-4)GlcNAcβ(1-3)Galβ(1-4)GlcNAcβ(1-3)Galβ(1-4)GlcNAcβ(1-3)GalNAcα-Thr-NH2	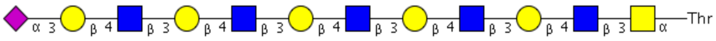
44	NeuAcα(2-3)Galβ(1-4)GlcNAcβ(1-3)[NeuAcα(2-3)Galβ(1-4)GlcNAcβ(1-6)]-GalNAcα-Thr-NH2	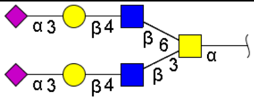
45	NeuAcα(2-3)Galβ(1-4)GlcNAcβ(1-3)Galβ(1-4)GlcNAcβ(1-3)[NeuAcα(2-3)Galβ(1-4)GlcNAcβ(1-3)Galβ(1-4)GlcNAcβ(1-6)]-GalNAcα-Thr-NH2	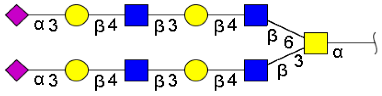
46	NeuAcα(2-3)Galβ(1-4)GlcNAcβ(1-3)Galβ(1-4)GlcNAcβ(1-3)Galβ(1-4)GlcNAcβ(1-3)[NeuAcα(2-3)Galβ(1-4)GlcNAcβ(1-3)Galβ(1-4)GlcNAcβ(1-3)Galβ(1-4)GlcNAcβ(1-6)]-GalNAcα-Thr-NH2	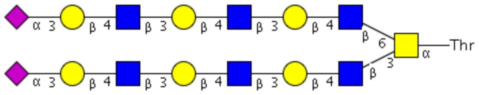
47	NeuAcα(2-3)Galβ(1-4)GlcNAcβ(1-3)Galβ(1-4)GlcNAcβ(1-3)Galβ(1-4)GlcNAcβ(1-3)Galβ(1-4)GlcNAcβ(1-3)[NeuAcα(2-3)Galβ(1-4)GlcNAcβ(1-3)Galβ(1-4)GlcNAcβ(1-3)Galβ(1-4)GlcNAcβ(1-3)Galβ(1-4)GlcNAcβ(1-6)]-GalNAcα-Thr-NH2	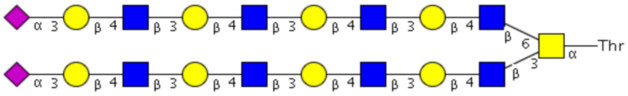
48	NeuAcα(2-3)Galβ(1-4)GlcNAcβ(1-3)Galβ(1-4)GlcNAcβ(1-3)Galβ(1-4)GlcNAcβ(1-3)Galβ(1-4)GlcNAcβ(1-3)Galβ(1-4)GlcNAcβ(1-3)[NeuAcα(2-3)Galβ(1-4)GlcNAcβ(1-3)Galβ(1-4)GlcNAcβ(1-3)Galβ(1-4)GlcNAcβ(1-3)Galβ(1-4)GlcNAcβ(1-3)Galβ(1-4)GlcNAcβ(1-6)]-GalNAcα-Thr-NH2	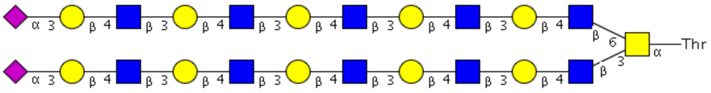

Glycan #	Common Name	Structure
49	NeuAcα(2-3)Galβ(1-4)GlcNAcβ(1-3)Galβ(1-4)GlcNAcβ(1-3)Galβ(1-4)GlcNAcβ(1-3)Galβ(1-4)GlcNAcβ(1-6)GalNAcα-Thr-NH2	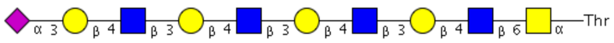
50	NeuAcα(2-3)Galβ(1-4)GlcNAcβ(1-3)Galβ(1-4)GlcNAcβ(1-3)Galβ(1-4)GlcNAcβ(1-3)Galβ(1-4)GlcNAcβ(1-3)Galβ(1-4)GlcNAcβ(1-6)GalNAcα-Thr-NH2	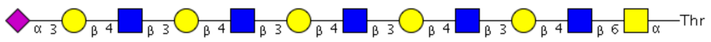
51	NeuAcα(2-3)Galβ(1-3)GlcNAcβ(1-3)Galβ(1-4)GlcNAcβ(1-6)[NeuAcα(2-3)Galβ(1-3)GlcNAcβ(1-3)] Galβ(1-4)GlcNAcβ-ethyl-NH2	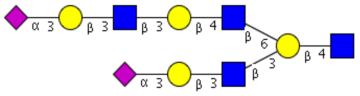
52	NeuAcα(2-3)Galβ(1-4)GlcNAcβ(1-3)Galβ(1-4)GlcNAcβ(1-3)Galβ(1-4)GlcNAcβ(1-6)[NeuAcα(2-3)Galβ(1-4)GlcNAcβ(1-3)Galβ(1-4)GlcNAcβ(1-3)] Galβ(1-4)GlcNAcβ-ethyl-NH2	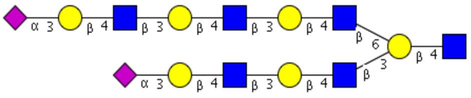
53	NeuAcα(2-3)Galβ(1-4)GlcNAcβ(1-2)Manα(1-3)[NeuAcα(2-3)Galβ(1-4)GlcNAcβ(1-2)Manα(1-6)]-Manβ(1-4)GlcNAcβ(1-4)GlcNAcβ-Asn-NH2	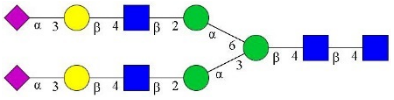
54	NeuAcα(2-3)Galβ(1-4)GlcNAcβ(1-3)Galβ(1-4)GlcNAcβ(1-2)Manα(1-3)[NeuAcα(2-3)Galβ(1-4)GlcNAcβ(1-3)Galβ(1-4)GlcNAcβ(1-2)Manα(1-6)]-Manβ(1-4)GlcNAcβ(1-4)GlcNAcβ-Asn-NH2	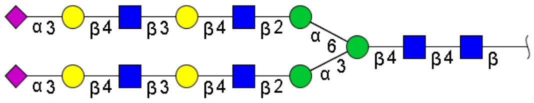
55	NeuAcα(2-3)Galβ(1-4)GlcNAcβ(1-3)Galβ(1-4)GlcNAcβ(1-3)Galβ(1-4)GlcNAcβ(1-2)Manα(1-3)[NeuAcα(2-3)Galβ(1-4)GlcNAcβ(1-3)Galβ(1-4)GlcNAcβ(1-3)Galβ(1-4)GlcNAcβ(1-2)Manα(1-6)]-Manβ(1-4)GlcNAcβ(1-4)GlcNAcβ-Asn-NH2	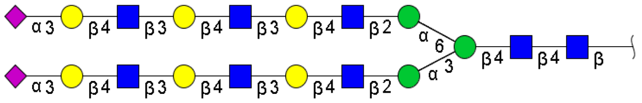
56	NeuAcα(2-3)Galβ(1-4)GlcNAcβ(1-3)Galβ(1-4)GlcNAcβ(1-2)Manα(1-3)[NeuAcα(2-3)Galβ(1-4)GlcNAcβ(1-3)Galβ(1-4)GlcNAcβ(1-2)Manα(1-6)]-Manβ(1-4)GlcNAcβ(1-4)GlcNAcβ-(Lys-Val-Ala)Asn-Lys-Thr-NH2	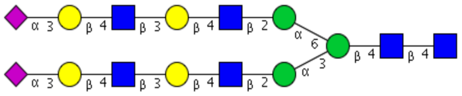
57	NeuAcα(2-3)Galβ(1-4)GlcNAcβ(1-3)Galβ(1-4)GlcNAcβ(1-3)Galβ(1-4)GlcNAcβ(1-2)Manα(1-3)[NeuAcα(2-3)Galβ(1-4)GlcNAcβ(1-3)Galβ(1-4)GlcNAcβ(1-3)Galβ(1-4)GlcNAcβ(1-2)Manα(1-6)]-Manβ(1-4)GlcNAcβ(1-4)GlcNAcβ-(Lys-Val-Ala)Asn-Lys-Thr-NH2	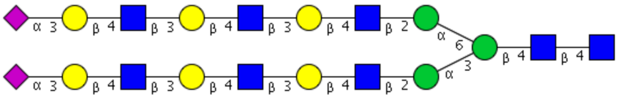
58	NeuAcα(2-3)Galβ(1-4)GlcNAcβ(1-3)Galβ(1-4)GlcNAcβ(1-3)Galβ(1-4)GlcNAcβ(1-3)Galβ(1-4)GlcNAcβ(1-2)Manα(1-3)[NeuAcα(2-3)Galβ(1-4)GlcNAcβ(1-3)Galβ(1-4)GlcNAcβ(1-3)Galβ(1-4)GlcNAcβ(1-3)Galβ(1-4)GlcNAcβ(1-2)Manα(1-6)]-Manβ(1-4)GlcNAcβ(1-4)GlcNAcβ-(Lys-Val-Ala)Asn-Lys-Thr-NH2	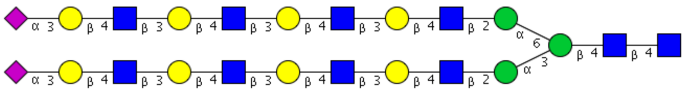

Glycan #	Common Name	Structure
59	NeuAcα(2-3)Galβ(1-4)GlcNAcβ(1-3)Galβ(1-4)GlcNAcβ(1-3)Galβ(1-4)GlcNAcβ(1-3)Galβ(1-4)GlcNAcβ(1-3)Galβ(1-4)GlcNAcβ(1-2)Manα(1-3)[NeuAcα(2-3)Galβ(1-4)GlcNAcβ(1-3)Galβ(1-4)GlcNAcβ(1-3)Galβ(1-4)GlcNAcβ(1-3)Galβ(1-4)GlcNAcβ(1-3)Galβ(1-4)GlcNAcβ(1-2)Manα(1-6)]-Manβ(1-4)GlcNAcβ(1-4)GlcNAcβ-(Lys-Val-Ala)Asn-Lys-Thr-NH2	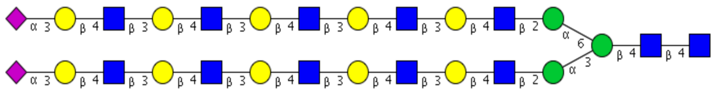
60	NeuAcα(2-3)Galβ(1-4)GlcNAcβ(1-3)Galβ(1-4)GlcNAcβ(1-3)Galβ(1-4)GlcNAcβ(1-2)Manα(1-3)[NeuAcα(2-3)Galβ(1-4)GlcNAcβ(1-3)Galβ(1-4)GlcNAcβ(1-3)Galβ(1-4)GlcNAcβ(1-2)Manα(1-6)]-Manβ(1-4)GlcNAcβ(1-4)[Fucα(1-6)]-GlcNAcβ-(Lys-Val-Ala)Asn-Lys-Thr-NH2	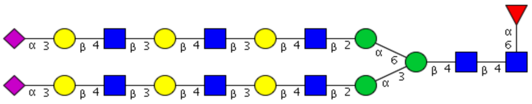
61	NeuAcα(2-3)Galβ(1-4)GlcNAcβ(1-3)Galβ(1-4)GlcNAcβ(1-3)Galβ(1-4)GlcNAcβ(1-3)Galβ(1-4)GlcNAcβ(1-2)Manα(1-3)[NeuAcα(2-3)Galβ(1-4)GlcNAcβ(1-3)Galβ(1-4)GlcNAcβ(1-3)Galβ(1-4)GlcNAcβ(1-3)Galβ(1-4)GlcNAcβ(1-2)Manα(1-6)]-Manβ(1-4)GlcNAcβ(1-4)[Fucα(1-6)]-GlcNAcβ-(Lys-Val-Ala)Asn-Lys-Thr-NH2	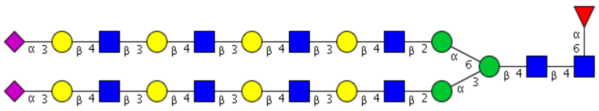
62	NeuAcα(2-3)Galβ(1-4)GlcNAcβ(1-3)Galβ(1-4)GlcNAcβ(1-2)Manα(1-3){NeuAcα(2-3)Galβ(1-4)GlcNAcβ(1-3)Galβ(1-4)GlcNAcβ(1-2)[NeuAcα(2-3)Galβ(1-4)GlcNAcβ(1-3)Galβ(1-4)GlcNAcβ(1-6)Manα(1-6)]}-Manβ(1-4)GlcNAcβ(1-4)GlcNAcβ-(Lys-Val-Ala)Asn-Lys-Thr-NH2	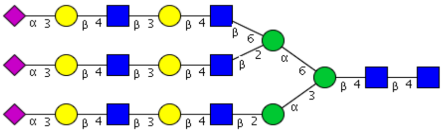
63	NeuAcα(2-3)Galβ(1-4)GlcNAcβ(1-3)Galβ(1-4)GlcNAcβ(1-3)Galβ(1-4)GlcNAcβ(1-2)Manα(1-3){NeuAcα(2-3)Galβ(1-4)GlcNAcβ(1-3)Galβ(1-4)GlcNAcβ(1-3)Galβ(1-4)GlcNAcβ(1-2)[NeuAcα(2-3)Galβ(1-4)GlcNAcβ(1-3)Galβ(1-4)GlcNAcβ(1-3)Galβ(1-4)GlcNAcβ(1-6)Manα(1-6)]}-Manβ(1-4)GlcNAcβ(1-4)GlcNAcβ-(Lys-Val-Ala)Asn-Lys-Thr-NH2	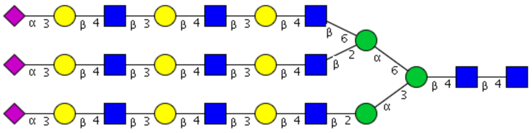
64	NeuAcα(2-3)Galβ(1-4)GlcNAcβ(1-3)Galβ(1-4)GlcNAcβ(1-3)Galβ(1-4)GlcNAcβ(1-3)Galβ(1-4)GlcNAcβ(1-2)Manα(1-3){NeuAcα(2-3)Galβ(1-4)GlcNAcβ(1-3)Galβ(1-4)GlcNAcβ(1-3)Galβ(1-4)GlcNAcβ(1-3)Galβ(1-4)GlcNAcβ(1-2)[NeuAcα(2-3)Galβ(1-4)GlcNAcβ(1-3)Galβ(1-4)GlcNAcβ(1-3)Galβ(1-4)GlcNAcβ(1-3)Galβ(1-4)GlcNAcβ(1-6)Manα(1-6)]}-Manβ(1-4)GlcNAcβ(1-4)GlcNAcβ-(Lys-Val-Ala)Asn-Lys-Thr-NH2	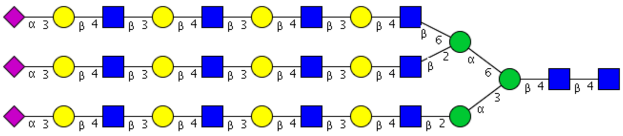

Glycan #	Common Name	Structure
65	NeuAcα(2-3)Galβ(1-4)GlcNAcβ(1-3)Galβ(1-4)GlcNAcβ(1-2)Manα(1-3){NeuAcα(2-3)Galβ(1-4)GlcNAcβ(1-3)Galβ(1-4)GlcNAcβ(1-2)[NeuAcα(2-3)Galβ(1-4)GlcNAcβ(1-3)Galβ(1-4)GlcNAcβ(1-6)Manα(1-6)]}-Manβ(1-4)GlcNAcβ(1-4)[Fucα(1-6)]-GlcNAcβ-(Lys-Val-Ala)Asn-Lys-Thr-NH2	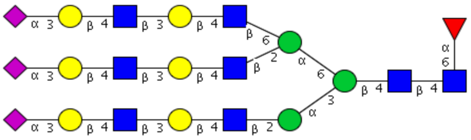
66	NeuAcα(2-3)Galβ(1-4)GlcNAcβ(1-3)Galβ(1-4)GlcNAcβ(1-3)Galβ(1-4)GlcNAcβ(1-2)Manα(1-3)-Manβ(1-4)GlcNAcβ(1-4)[Fucα(1-6)]-GlcNAcβ-(Lys-Val-Ala)Asn-Lys-Thr-NH2	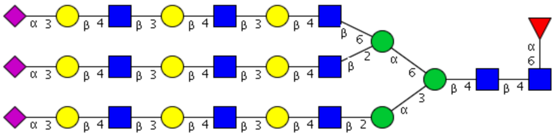
67	NeuAcα(2-3)Galβ(1-4)GlcNAcβ(1-3)Galβ(1-4)GlcNAcβ(1-3)Galβ(1-4)GlcNAcβ(1-3)Galβ(1-4)GlcNAcβ(1-2)Manα(1-3){NeuAcα(2-3)Galβ(1-4)GlcNAcβ(1-3)Galβ(1-4)GlcNAcβ(1-3)Galβ(1-4)GlcNAcβ(1-3)Galβ(1-4)GlcNAcβ(1-2)[NeuAcα(2-3)Galβ(1-4)GlcNAcβ(1-3)Galβ(1-4)GlcNAcβ(1-3)Galβ(1-4)GlcNAcβ(1-3)Galβ(1-4)GlcNAcβ(1-6)Manα(1-6)]}-Manβ(1-4)GlcNAcβ(1-4)[Fucα(1-6)]-GlcNAcβ-(Lys-Val-Ala)Asn-Lys-Thr-NH2	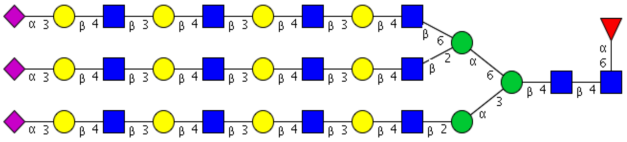
68	Gn/3'SLN/3'SLN-TriN	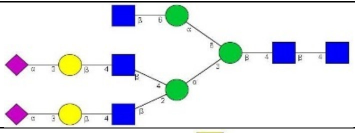
69	NeuAcα(2-3)[GalNAcβ(1-4)]-Galβ(1-4)GlcNAcβ-ethyl-NH2	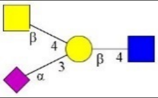
70	NeuAcα(2-3)[GalNAcβ(1-4)]-Galβ(1-4)Glcβ-ethyl-NH2	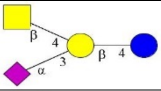
71	Galβ(1-3)GalNAcβ(1-4)[NeuAcα(2-3)]-Galβ(1-4)Glcβ-ethyl-NH2	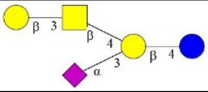
72	NeuAcα(2-3)Galβ(1-4)[Fucα(1-3)]-GlcNAcβ-propyl-NH2	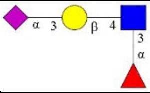
73	NeuAcα(2-3)Galβ(1-3)[Fucα(1-4)]-GlcNAcβ(1-3)Galβ(1-4)[Fucα(1-3)]-GlcNAcβ-ethyl-NH2	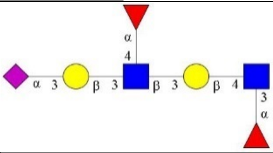
74	NeuAcα(2-3)Galβ(1-4)[Fucα(1-3)]-GlcNAcβ(1-3)Galβ(1-4)[Fucα(1-3)]-GlcNAcβ-ethyl-NH2	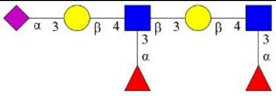
75	NeuAcα(2-3)Galβ(1-4)[Fucα(1-3)]-GlcNAcβ(1-3)Galβ(1-4)[Fucα(1-3)]-GlcNAcβ(1-3)Galβ(1-4)[Fucα(1-3)]-GlcNAcβ-ethyl-NH2	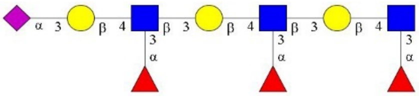

Glycan #	Common Name	Structure
76	NeuAcα(2-3)Galβ(1-4)[Fucα(1-3)]-GlcNAcβ(1-3)Galβ(1-4)[Fucα(1-3)]-GlcNAcβ(1-3)Galβ(1-4)[Fucα(1-3)]-GlcNAcβ(1-3)Galβ(1-3)GalNAcα-Thr-NH2	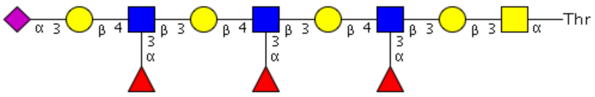
77	NeuAcα(2-3)Galβ(1-4)[Fucα(1-3)]-GlcNAcβ(1-3)Galβ(1-4)[Fucα(1-3)]-GlcNAcβ(1-3)Galβ(1-4)[Fucα(1-3)]-GlcNAcβ(1-3)GalNAcα-Thr-NH2	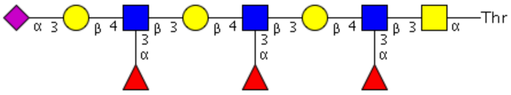
78	NeuAcα(2-3)Galβ(1-4)[Fucα(1-3)]-GlcNAcβ(1-3)Galβ(1-4)[Fucα(1-3)]-GlcNAcβ(1-3)Galβ(1-4)[Fucα(1-3)]-GlcNAcβ(1-3)[NeuAcα(2-3)Galβ(1-4)[Fucα(1-3)]-GlcNAcβ(1-3)Galβ(1-4)[Fucα(1-3)]-GlcNAcβ(1-3)Galβ(1-4)[Fucα(1-3)]-GlcNAcβ(1-6)]-GalNAcα-Thr-NH2	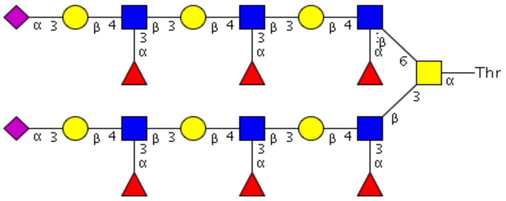
79	NeuAcα(2-3)Galβ(1-4)[Fucα(1-3)]-GlcNAcβ(1-2)Manα(1-3)[NeuAcα(2-3)Galβ(1-4)[Fucα(1-3)]-GlcNAcβ(1-2)Manα(1-6)]-Manβ(1-4)GlcNAcβ(1-4)GlcNAcβ-(Lys-Val-Ala)Asn-(Lys-Thr)NH2	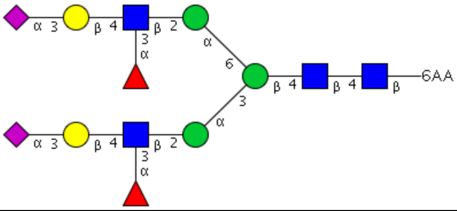
80	NeuAcα(2-6)Galb(1-4)(6S)GlcNacβ-ethyl-NH2	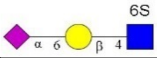
81	NeuAcα(2-6)Galβ(1-4)6-O-sulfo-GlcNAcβ-propyl-NH2	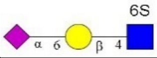
82	NeuAcα(2-6)Galβ(1-4)Glcβ-ethyl-NH2	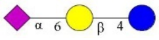
83	NeuAcα(2-6)Galβ(1-4)GlcNAcβ-ethyl-NH2	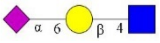
84	NeuAcα(2-6)Galβ(1-4)GlcNAcβ(1-3)Galβ(1-4)GlcNAcβ-ethyl-NH2	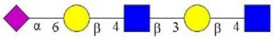
85	NeuAcα(2-6)Galβ(1-4)GlcNAcβ(1-3)Galβ(1-4)GlcNAcβ(1-3)Galβ(1-4)GlcNAcβ-ethyl-NH2	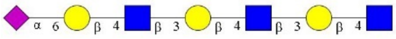
86	NeuAcα(2-6)GalNAcβ(1-4)GlcNAcβ-ethyl-NH2	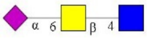
87	NeuAcα(2-6)Galβ(1-4)GlcNAcβ(1-3)Galβ(1-3)GalNAcα-Thr-NH2	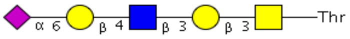
88	NeuAcα(2-6)Galβ(1-4)GlcNAcβ(1-3)Galβ(1-4)GlcNAcβ(1-3)Galβ(1-3)GalNAcα-Thr-NH2	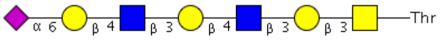
89	NeuAcα(2-6)Galβ(1-4)GlcNAcβ(1-3)Galβ(1-4)GlcNAcβ(1-3)Galβ(1-4)GlcNAcβ(1-3)Galβ(1-3)GalNAcα-Thr-NH2	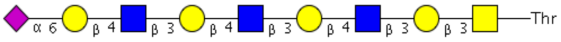
90	NeuAcα(2-6)Galβ(1-4)GlcNAcβ(1-3)Galβ(1-4)GlcNAcβ(1-3)Galβ(1-4)GlcNAcβ(1-3)Galβ(1-4)GlcNAcβ(1-3)Galβ(1-3)GalNAcα-Thr-NH2	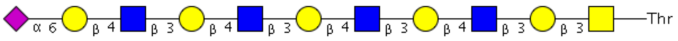
91	NeuAcα(2-6)Galβ(1-4)GlcNAcβ(1-3)Galβ(1-4)GlcNAcβ(1-3)Galβ(1-4)GlcNAcβ(1-3)Galβ(1-4)GlcNAcβ(1-3)Galβ(1-4)GlcNAcβ(1-3)Galβ(1-3)GalNAcα-Thr-NH2	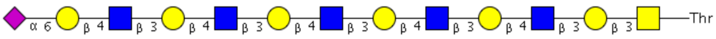
92	NeuAcα(2-6)Galβ(1-4)GlcNAcβ(1-6)[Galβ(1-3)]-GalNAcα-Thr-NH2	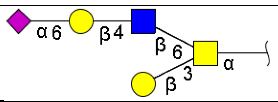
93	NeuAcα(2-6)Galβ(1-4)GlcNAcβ(1-3)Galβ(1-4)GlcNAcβ(1-6)[Galβ(1-3)]-GalNAcα-Thr-NH2	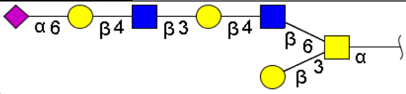

Glycan #	Common Name	Structure
94	NeuAcα(2-6)Galβ(1-4)GlcNAcβ(1-3)Galβ(1-4)GlcNAcβ(1-3)Galβ(1-4)GlcNAcβ(1-6)[Galβ(1-3)]-GalNAcα-Thr-NH2	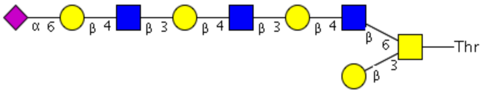
95	NeuAcα(2-6)Galβ(1-4)GlcNAcβ(1-3)Galβ(1-4)GlcNAcβ(1-3)Galβ(1-4)GlcNAcβ(1-3)Galβ(1-4)GlcNAcβ(1-6)[Galβ(1-3)]-GalNAcα-Thr-NH2	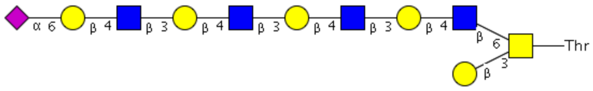
96	NeuAcα(2-6)Galβ(1-4)GlcNAcβ(1-3)Galβ(1-4)GlcNAcβ(1-3)Galβ(1-4)GlcNAcβ(1-3)Galβ(1-4)GlcNAcβ(1-3)Galβ(1-4)GlcNAcβ(1-6)[Galβ(1-3)]-GalNAcα-Thr-NH2	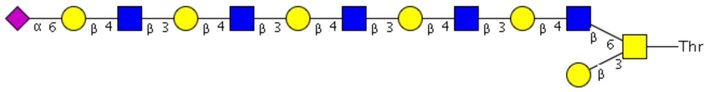
97	NeuAcα(2-6)Galβ(1-4)GlcNAcβ(1-3)Galβ(1-4)GlcNAcβ(1-3)Galβ(1-4)GlcNAcβ(1-3)Galβ(1-4)GlcNAcβ(1-6)[NeuAcα(2-6)Galβ(1-4)GlcNAcβ(1-3)Galβ(1-4)GlcNAcβ(1-3)Galβ(1-4)GlcNAcβ(1-3)Galβ(1-3)]-GalNAcα-Thr-NH2	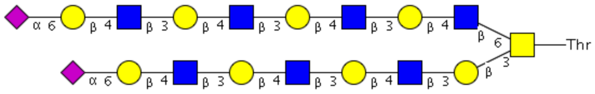
98	NeuAcα(2-6)Galβ(1-4)GlcNAcβ(1-3)Galβ(1-4)GlcNAcβ(1-3)Galβ(1-4)GlcNAcβ(1-3)Galβ(1-4)GlcNAcβ(1-3)Galβ(1-4)GlcNAcβ(1-6)[NeuAcα(2-6)Galβ(1-4)GlcNAcβ(1-3)Galβ(1-4)GlcNAcβ(1-3)Galβ(1-4)GlcNAcβ(1-3)Galβ(1-4)GlcNAcβ(1-3)Galβ(1-3)]-GalNAcα-Thr-NH2	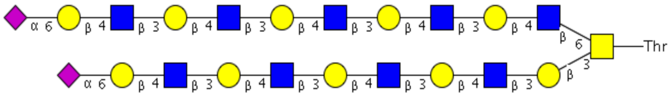
99	NeuAcα(2-6)Galβ(1-4)GlcNAcβ(1-3)GalNAcα-Thr-NH2	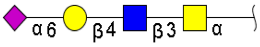
100	NeuAcα(2-6)Galβ(1-4)GlcNAcβ(1-3)Galβ(1-4)GlcNAcβ(1-3)GalNAcα-Thr-NH2	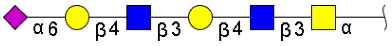
101	NeuAcα(2-6)Galβ(1-4)GlcNAcβ(1-3)Galβ(1-4)GlcNAcβ(1-3)Galβ(1-4)GlcNAcβ(1-3)GalNAcα-Thr-NH2	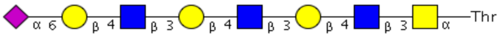
102	NeuAcα(2-6)Galβ(1-4)GlcNAcβ(1-3)Galβ(1-4)GlcNAcβ(1-3)Galβ(1-4)GlcNAcβ(1-3)Galβ(1-4)GlcNAcβ(1-3)GalNAcα-Thr-NH2	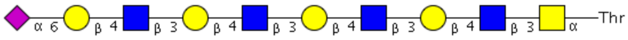
103	NeuAcα(2-6)Galβ(1-4)GlcNAcβ(1-3)Galβ(1-4)GlcNAcβ(1-3)Galβ(1-4)GlcNAcβ(1-3)Galβ(1-4)GlcNAcβ(1-3)Galβ(1-4)GlcNAcβ(1-3)GalNAcα-Thr-NH2	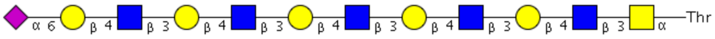
104	NeuAcα(2-6)Galβ(1-4)GlcNAcβ(1-3)[NeuAcα(2-6)Galβ(1-4)GlcNAcβ(1-6)]-GalNAcα-Thr-NH2	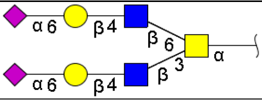
105	NeuAcα(2-6)Galβ(1-4)GlcNAcβ(1-3)Galβ(1-4)GlcNAcβ(1-3)[NeuAcα(2-6)Galβ(1-4)GlcNAcβ(1-3)Galβ(1-4)GlcNAcβ(1-6)]-GalNAcα-Thr-NH2	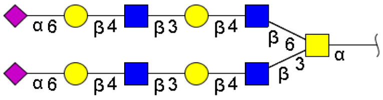
106	NeuAcα(2-6)Galβ(1-4)GlcNAcβ(1-3)Galβ(1-4)GlcNAcβ(1-3)[NeuAcα(2-6)Galβ(1-4)GlcNAcβ(1-3)Galβ(1-4)GlcNAcβ(1-3)Galβ(1-4)GlcNAcβ(1-6)]-GalNAcα-Thr-NH2	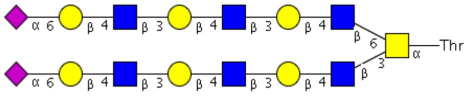

Glycan #	Common Name	Structure
107	NeuAcα(2-6)Galβ(1-4)GlcNAcβ(1-3)Galβ(1-4)GlcNAcβ(1-3)Galβ(1-4)GlcNAcβ(1-3)Galβ(1-4)GlcNAcβ(1-3)[NeuAcα(2-6)Galβ(1-4)GlcNAcβ(1-3)Galβ(1-4)GlcNAcβ(1-3)Galβ(1-4)GlcNAcβ(1-3)Galβ(1-4)GlcNAcβ(1-6)]-GalNAcα-Thr-NH2	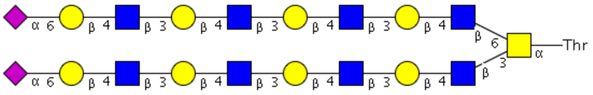
108	NeuAcα(2-6)Galβ(1-4)GlcNAcβ(1-3)Galβ(1-4)GlcNAcβ(1-3)Galβ(1-4)GlcNAcβ(1-3)Galβ(1-4)GlcNAcβ(1-3)Galβ(1-4)GlcNAcβ(1-3)[NeuAcα(2-6)Galβ(1-4)GlcNAcβ(1-3)Galβ(1-4)GlcNAcβ(1-3)Galβ(1-4)GlcNAcβ(1-3)Galβ(1-4)GlcNAcβ(1-3)Galβ(1-4)GlcNAcβ(1-6)]-GalNAcα-Thr-NH2	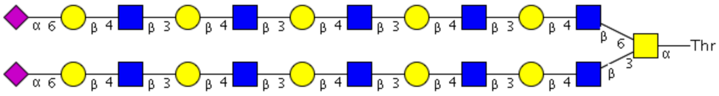
109	NeuAcα(2-6)Galβ(1-4)GlcNAcβ(1-3)Galβ(1-4)GlcNAcβ(1-3)Galβ(1-4)GlcNAcβ(1-3)Galβ(1-4)GlcNAcβ(1-6)GalNAcα-Thr-NH2	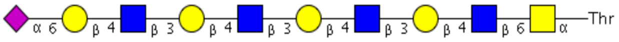
110	NeuAcα(2-6)Galβ(1-4)GlcNAcβ(1-3)Galβ(1-4)GlcNAcβ(1-3)Galβ(1-4)GlcNAcβ(1-3)Galβ(1-4)GlcNAcβ(1-3)Galβ(1-4)GlcNAcβ(1-6)GalNAcα-Thr-NH2	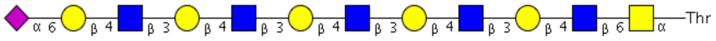
111	NeuAcα(2-6)Galβ(1-4)GlcNAcβ(1-3)Galβ(1-4)GlcNAcβ(1-3)Galβ(1-4)GlcNAcβ(1-6)[NeuAcα(2-6)Galβ(1-4)GlcNAcβ(1-3)Galβ(1-4)GlcNAcβ(1-3)] Galβ(1-4)GlcNAcβ-ethyl-NH2	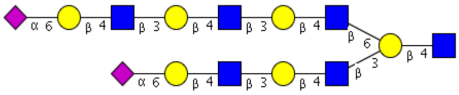
112	NeuAcα(2-6)Galβ(1-3)GlcNAcβ(1-3)Galβ(1-4)GlcNAcβ(1-6)[NeuAcα(2-6)Galβ(1-3)GlcNAcβ(1-3)] Galβ(1-4)GlcNAcβ-ethyl-NH2	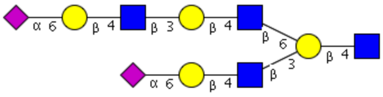
113	Galβ(1-4)GlcNAcβ(1-2)Manα(1-3)[NeuAcα(2-6)Galβ(1-4)GlcNAcβ(1-2)Manα(1-6)]-Manβ(1-4)GlcNAcβ(1-4)GlcNAcβ-Asn-NH2	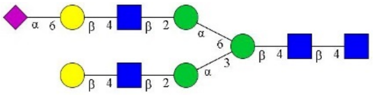
114	NeuAcα(2-6)Galβ(1-4)GlcNAcβ(1-2)Manα(1-3)[Galβ(1-4)GlcNAcβ(1-2)Manα(1-6)]-Manβ(1-4)GlcNAcβ(1-4)GlcNAcβ-Asn-NH2	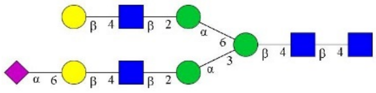
115	GlcNAcβ(1-2)Manα(1-3)[NeuAcα(2-6)Galβ(1-4)GlcNAcβ(1-2)Manα(1-6)]-Manβ(1-4)GlcNAcβ(1-4)GlcNAcβ-Asn-NH2	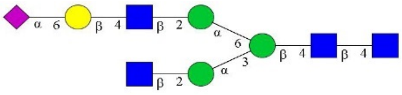
116	NeuAcα(2-6)Galβ(1-4)GlcNAcβ(1-2)Manα(1-3)[NeuAcα(2-6)Galβ(1-4)GlcNAcβ(1-2)Manα(1-6)]-Manβ(1-4)GlcNAcβ(1-4)GlcNAcβ-Asn-NH2	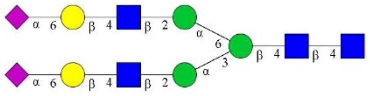
117	NeuAcα(2-6)Galβ(1-4)GlcNAcβ(1-3)Galβ(1-4)GlcNAcβ(1-2)Manα(1-3)[NeuAcα(2-6)Galβ(1-4)GlcNAcβ(1-3)Galβ(1-4)GlcNAcβ(1-2)Manα(1-6)]-Manβ(1-4)GlcNAcβ(1-4)GlcNAcβ-Asn-NH2	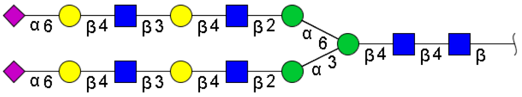
118	NeuAcα(2-6)Galβ(1-4)GlcNAcβ(1-3)Galβ(1-4)GlcNAcβ(1-2)Manα(1-3)[NeuAcα(2-6)Galβ(1-4)GlcNAcβ(1-3)Galβ(1-4)GlcNAcβ(1-2)Manα(1-6)]-Manβ(1-4)GlcNAcβ(1-4)GlcNAcβ-(Lys-Val-Ala)Asn-Lys-Thr-NH2	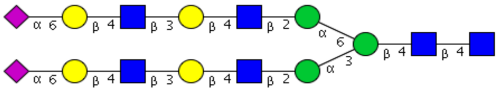

Glycan #	Common Name	Structure
119	NeuAcα(2-6)Galβ(1-4)GlcNAcβ(1-3)Galβ(1-4)GlcNAcβ(1-3)Galβ(1-4)GlcNAcβ(1-2)Manα(1-3)[NeuAcα(2-6)Galβ(1-4)GlcNAcβ(1-3)Galβ(1-4)GlcNAcβ(1-3)Galβ(1-4)GlcNAcβ(1-2)Manα(1-6)]-Manβ(1-4)GlcNAcβ(1-4)GlcNAcβ-Asn-NH2	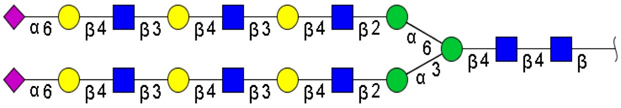
120	NeuAcα(2-6)Galβ(1-4)GlcNAcβ(1-3)Galβ(1-4)GlcNAcβ(1-3)Galβ(1-4)GlcNAcβ(1-2)Manα(1-3)[NeuAcα(2-6)Galβ(1-4)GlcNAcβ(1-3)Galβ(1-4)GlcNAcβ(1-3)Galβ(1-4)GlcNAcβ(1-2)Manα(1-6)]-Manβ(1-4)GlcNAcβ(1-4)GlcNAcβ-(Lys-Val-Ala)Asn-Lys-Thr-NH2	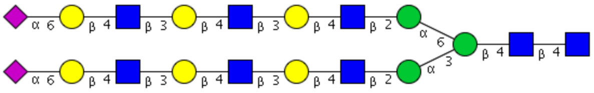
121	NeuAcα(2-6)Galβ(1-4)GlcNAcβ(1-3)Galβ(1-4)GlcNAcβ(1-3)Galβ(1-4)GlcNAcβ(1-3)Galβ(1-4)GlcNAcβ(1-2)Manα(1-3)[NeuAcα(2-6)Galβ(1-4)GlcNAcβ(1-3)Galβ(1-4)GlcNAcβ(1-3)Galβ(1-4)GlcNAcβ(1-3)Galβ(1-4)GlcNAcβ(1-2)Manα(1-6)]-Manβ(1-4)GlcNAcβ(1-4)GlcNAcβ-(Lys-Val-Ala)Asn-Lys-Thr-NH2	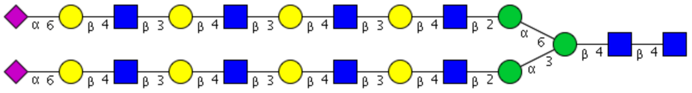
122	NeuAcα(2-6)Galβ(1-4)GlcNAcβ(1-3)Galβ(1-4)GlcNAcβ(1-3)Galβ(1-4)GlcNAcβ(1-3)Galβ(1-4)GlcNAcβ(1-3)Galβ(1-4)GlcNAcβ(1-2)Manα(1-3)[NeuAcα(2-6)Galβ(1-4)GlcNAcβ(1-3)Galβ(1-4)GlcNAcβ(1-3)Galβ(1-4)GlcNAcβ(1-3)Galβ(1-4)GlcNAcβ(1-3)Galβ(1-4)GlcNAcβ(1-2)Manα(1-6)]-Manβ(1-4)GlcNAcβ(1-4)GlcNAcβ-(Lys-Val-Ala)Asn-Lys-Thr-NH2	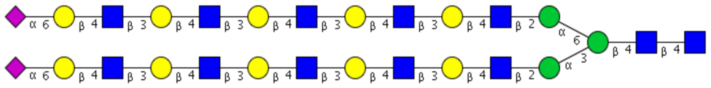
123	NeuAcα(2-6)Galβ(1-4)GlcNAcβ(1-3)Galβ(1-4)GlcNAcβ(1-2)Manα(1-3)[NeuAcα(2-6)Galβ(1-4)GlcNAcβ(1-3)Galβ(1-4)GlcNAcβ(1-2)Manα(1-6)]-Manβ(1-4)GlcNAcβ(1-4)[Fucα(1-6)]-GlcNAcβ-(Lys-Val-Ala)Asn-Lys-Thr-NH2	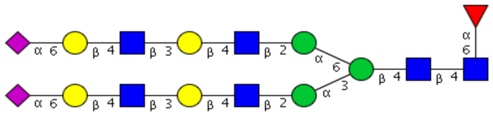
124	NeuAcα(2-6)Galβ(1-4)GlcNAcβ(1-3)Galβ(1-4)GlcNAcβ(1-3)Galβ(1-4)GlcNAcβ(1-2)Manα(1-3)[NeuAcα(2-6)Galβ(1-4)GlcNAcβ(1-3)Galβ(1-4)GlcNAcβ(1-3)Galβ(1-4)GlcNAcβ(1-2)Manα(1-6)]-Manβ(1-4)GlcNAcβ(1-4)[Fucα(1-6)]-GlcNAcβ-(Lys-Val-Ala)Asn-Lys-Thr-NH2	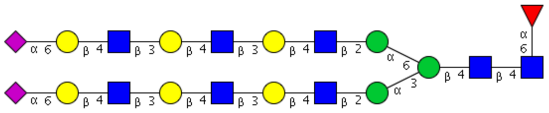

Glycan #	Common Name	Structure
125	NeuAcα(2-6)Galβ(1-4)GlcNAcβ(1-3)Galβ(1-4)GlcNAcβ(1-3)Galβ(1-4)GlcNAcβ(1-3)Galβ(1-4)GlcNAcβ(1-2)Manα(1-3)[NeuAcα(2-6)Galβ(1-4)GlcNAcβ(1-3)Galβ(1-4)GlcNAcβ(1-3)Galβ(1-4)GlcNAcβ(1-3)Galβ(1-4)GlcNAcβ(1-2)Manα(1-6)]-Manβ(1-4)GlcNAcβ(1-4)[Fucα(1-6)]-GlcNAcβ-(Lys-Val-Ala)Asn-Lys-Thr-NH2	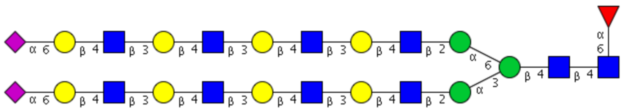
126	NeuAcα(2-6)Galβ(1-4)GlcNAcβ(1-3)Galβ(1-4)GlcNAcβ(1-2)Manα(1-3){NeuAcα(2-6)Galβ(1-4)GlcNAcβ(1-3)Galβ(1-4)GlcNAcβ(1-2)[NeuAcα(2-6)Galβ(1-4)GlcNAcβ(1-3)Galβ(1-4)GlcNAcβ(1-6)Manα(1-6)]}-Manβ(1-4)GlcNAcβ(1-4)GlcNAcβ-(Lys-Val-Ala)Asn-Lys-Thr-NH2	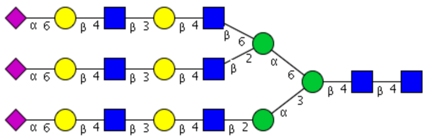
127	NeuAcα(2-6)Galβ(1-4)GlcNAcβ(1-3)Galβ(1-4)GlcNAcβ(1-3)Galβ(1-4)GlcNAcβ(1-2)Manα(1-3){NeuAcα(2-6)Galβ(1-4)GlcNAcβ(1-3)Galβ(1-4)GlcNAcβ(1-3)Galβ(1-4)GlcNAcβ(1-2)[NeuAcα(2-6)Galβ(1-4)GlcNAcβ(1-3)Galβ(1-4)GlcNAcβ(1-3)Galβ(1-4)GlcNAcβ(1-6)Manα(1-6)]}-Manβ(1-4)GlcNAcβ(1-4)GlcNAcβ-(Lys-Val-Ala)Asn-Lys-Thr-NH2	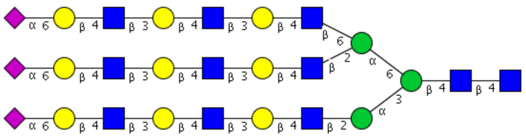
128	NeuAcα(2-6)Galβ(1-4)GlcNAcβ(1-3)Galβ(1-4)GlcNAcβ(1-3)Galβ(1-4)GlcNAcβ(1-3)Galβ(1-4)GlcNAcβ(1-2)Manα(1-3){NeuAcα(2-6)Galβ(1-4)GlcNAcβ(1-3)Galβ(1-4)GlcNAcβ(1-3)Galβ(1-4)GlcNAcβ(1-3)Galβ(1-4)GlcNAcβ(1-2)[NeuAcα(2-6)Galβ(1-4)GlcNAcβ(1-3)Galβ(1-4)GlcNAcβ(1-3)Galβ(1-4)GlcNAcβ(1-3)Galβ(1-4)GlcNAcβ(1-6)Manα(1-6)]}-Manβ(1-4)GlcNAcβ(1-4)GlcNAcβ-(Lys-Val-Ala)Asn-Lys-Thr-NH2	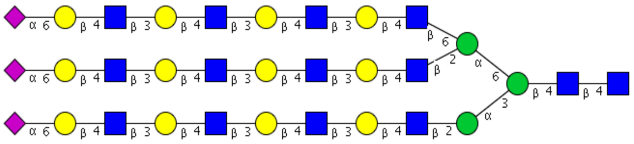
129	NeuAcα(2-6)Galβ(1-4)GlcNAcβ(1-3)Galβ(1-4)GlcNAcβ(1-2)Manα(1-3){NeuAcα(2-6)Galβ(1-4)GlcNAcβ(1-3)Galβ(1-4)GlcNAcβ(1-2)[NeuAcα(2-6)Galβ(1-4)GlcNAcβ(1-3)Galβ(1-4)GlcNAcβ(1-6)Manα(1-6)]}-Manβ(1-4)GlcNAcβ(1-4)[Fucα(1-6)]-GlcNAcβ-(Lys-Val-Ala)Asn-Lys-Thr-NH2	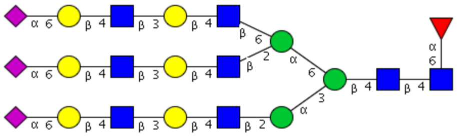

Glycan #	Common Name	Structure
130	NeuAcα(2-6)Galβ(1-4)GlcNAcβ(1-3)Galβ(1-4)GlcNAcβ(1-3)Galβ(1-4)GlcNAcβ(1-2)Manα(1-3){NeuAcα(2-6)Galβ(1-4)GlcNAcβ(1-3)Galβ(1-4)GlcNAcβ(1-3)Galβ(1-4)GlcNAcβ(1-2)[NeuAcα(2-6)Galβ(1-4)GlcNAcβ(1-3)Galβ(1-4)GlcNAcβ(1-3)Galβ(1-4)GlcNAcβ(1-6)Manα(1-6)]}-Manβ(1-4)GlcNAcβ(1-4)[Fucα(1-6)]-GlcNAcβ-(Lys-Val-Ala)Asn-Lys-Thr-NH2	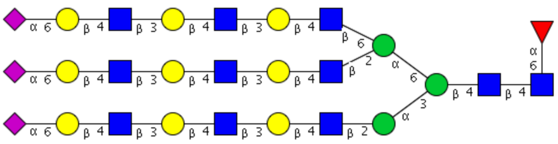
131	NeuAcα(2-6)Galβ(1-4)GlcNAcβ(1-3)Galβ(1-4)GlcNAcβ(1-3)Galβ(1-4)GlcNAcβ(1-3)Galβ(1-4)GlcNAcβ(1-2)Manα(1-3){NeuAcα(2-6)Galβ(1-4)GlcNAcβ(1-3)Galβ(1-4)GlcNAcβ(1-3)Galβ(1-4)GlcNAcβ(1-3)Galβ(1-4)GlcNAcβ(1-2)[NeuAcα(2-6)Galβ(1-4)GlcNAcβ(1-3)Galβ(1-4)GlcNAcβ(1-3)Galβ(1-4)GlcNAcβ(1-3)Galβ(1-4)GlcNAcβ(1-6)Manα(1-6)]}-Manβ(1-4)GlcNAcβ(1-4)[Fucα(1-6)]-GlcNAcβ-(Lys-Val-Ala)Asn-Lys-Thr-NH2	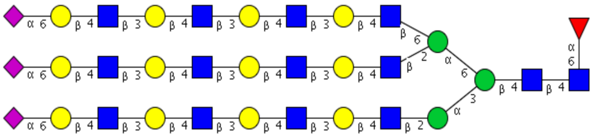
132	LN/6'SLN/6'SLN-TriN	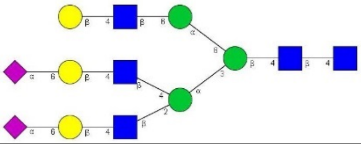
133	6'SLNLN/6'SLN/6'SLN-TriN	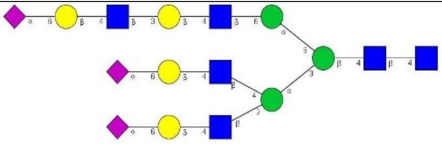
134	6'SLN/LeX/LeX-TriN	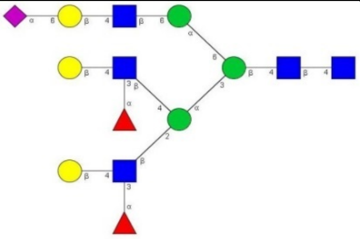
135	6'SLNLN/LeX/LeX-TriN	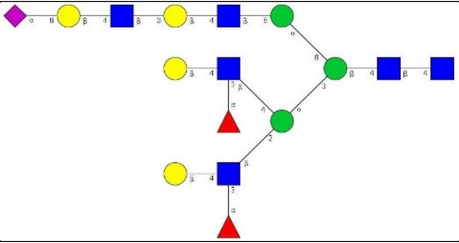
