# Supplementary material for: Three mutations switch H7N9 influenza to human-type receptor specificity
Source: PLoS Pathog. 2017 Jun 15;13(6):e1006390. doi: 10.1371/journal.ppat.1006390 (PMC5472306; doi:10.1371/journal.ppat.1006390)
Supplement: S2 Table — (PDF) [file ppat.1006390.s002.pdf]

S2 Table. Data collection and refinement statistics for the Sh2 H7 triple mutant (V186K, K193T, G228S)

Data set	*apo*	LSTc	LSTa
Space group	R32	R32	R32
Unit cell (Å)	*a* = *b* = 115.4, *c* = 294.9	*a* = *b* = 116.4, *c* = 296.0	*a* = *b* = 115.9, *c* = 295.8
Resolution (Å) [a]	50.0-2.60 (2.64-2.60)	50.0-2.60 (2.64-2.60)	50.0-2.90 (2.95-2.90)
X-ray source	APS 23ID-D	APS 23ID-D	APS 23ID-D
Unique reflections	23,692	24,049	17,020
Redundancy [a]	4.0 (2.8)	4.6 (3.1)	5.3 (4.1)
Average $I/\sigma(I)$ [a]	16.4 (1.0)	28.7 (1.0)	18.3 (1.2)
Completeness [a]	99.3 (93.4)	99.4 (96.7)	99.3 (97.7)
R_{sym} [a,b]	0.09 (0.66)	0.08 (0.70)	0.14 (0.93)
R_{pim} [a,b]	0.05 (0.44)	0.04 (0.44)	0.06 (0.50)
$CC_{1/2}$ [a,b]	0.998 (0.548)	0.998 (0.690)	0.996 (0.457)
HA protomers in a.u.	1	1	1
V_m (Å^3/Da)	3.3	3.4	3.4
Reflections in refinement	23,688	24,028	17,018
Refined residues	483	482	485
Refined waters	71	31	10
Refined ligand atoms	-	22	22
R_{cryst} [c]	0.203	0.218	0.201
R_{free} [d]	0.251	0.277	0.244
B-values (Å^2)			
Protein	84	99	100
Ligand	-	124	152
Waters	66	83	68
Wilson *B*-values (Å^2)	79	86	94
Ramachandran values (%) [e]	96.9, 0	96.2, 0.2	97.1, 0
r.m.s.d. bond (Å)	0.012	0.013	0.012
r.m.s.d. angle (deg.)	1.67	1.91	1.77
PDB codes	5VJK	5VJL	5VJM

[a] Parentheses denote outer-shell statistics.

[b] $R_{sym} = \sum_{hkl}\sum_i |I_{hkl,i} - \langle I_{hkl}\rangle| / \sum_{hkl}\sum_i I_{hkl,i}$ and $R_{pim} = \sum_{hkl}[1/(N-1)]^{1/2}\sum_i |I_{hkl,i} - \langle I_{hkl}\rangle| / \sum_{hkl}\sum_i I_{hkl,i}$, where $I_{hkl,i}$ is the scaled intensity of the ith measurement of reflection *h*, *k*, *l*, $\langle I_{hkl}\rangle$ is the average intensity for that reflection, and *N* is the redundancy. $R_{pim} = \Sigma_{hkl} (1/(n-1))^{1/2} \Sigma_i | I_{hkl,i} - \langle I_{hkl}\rangle | / \Sigma_{hkl} \Sigma_i I_{hkl,i}$, where n is the redundancy. $CC_{1/2}$ = Pearson Correlation Coefficient between two random half datasets.

[c] $R_{cryst} = \sum_{hkl} |F_o - F_c| / \sum_{hkl} |F_o|$, where F_o and F_c are the observed and calculated structure factors.

[d] R_{free} was calculated as for R_{cryst}, but on 5% of data excluded before refinement.

[e] The values are percentage of residues in the favored and outliers regions analyzed by MolProbity [1].

REFERENCES

1. Chen VB, Arendall WB, 3rd, Headd JJ, Keedy DA, Immormino RM, et al. (2010) MolProbity: all-atom structure validation for macromolecular crystallography. Acta Crystallogr D Biol Crystallogr 66: 12-21.
