# Supplementary material for: Three mutations switch H7N9 influenza to human-type receptor specificity
Source: PLoS Pathog. 2017 Jun 15;13(6):e1006390. doi: 10.1371/journal.ppat.1006390 (PMC5472306; doi:10.1371/journal.ppat.1006390)
Supplement: S3 Table — (PDF) [file ppat.1006390.s003.pdf]

S3 Table. Receptor binding of H7 mutants

HA	ELISA-like assay		Glycan array	
	α2-3	α2-6	α2-3	α2-6
WT	++[a]	-[b]	++	-
G225D	ND[c]	ND	-	-
E190D G225D	ND	ND	-	-
K193T	+[d]	-	-	-
V186K	-	+	-	-
V186K G228S	+	+	-	-
V186G	-	-	+	-
V186G G228S	+	-	+	+
V186G K193T	-	-	-	-
V186N	-	-	-	-
N224K	-	-	-	-
N224K G228S	++	++	++	-

[a] Significant binding above $1x10^4$ RFUs (see Fig. S2)

[b] Signals below $2x10^3$ RFUs

[c] Not determined

[d] Significant binding below $5x10^3$ RFUs
