# Supplementary material for: Three mutations switch H7N9 influenza to human-type receptor specificity
Source: PLoS Pathog. 2017 Jun 15;13(6):e1006390. doi: 10.1371/journal.ppat.1006390 (PMC5472306; doi:10.1371/journal.ppat.1006390)
Supplement: S4 Table — (PDF) [file ppat.1006390.s004.pdf]

S4 Table. Binding of Sh2 WT and Sh2 V186K-K193T-G228S variant HA to biotinylated α2-3 and α2-6 N-glycan receptors (quoted values are K_d apparent (μg ml^{-1}) ± standard error):

	Sh2	Sh2 G186K-K193T-G228S
**3SLN**	1.67 ± 0.16	n/b*
**$3SLN_2$**	2.59 ± 0.21	n/b*
**$3SLN_3$**	2.93 ± 0.23	n/b*
**$3SLN_4$**	2.37 ± 0.34	n/b*
**6SLN**	n/b*	n/b*
**$6SLN_2$**	48.08 ± 9.74#	55.50 ± 7.86#
**$6SLN_3$**	8.74 ± 0.51	3.82 ± 0.24
**$6SLN_4$**	21.16 ± 2.72#	4.68 ± 0.31

* n/b, no binding/insufficient to estimate K_d.
# K_d estimated via extrapolation to maximal binding.
