# Supplementary material for: Three mutations switch H7N9 influenza to human-type receptor specificity
Source: PLoS Pathog. 2017 Jun 15;13(6):e1006390. doi: 10.1371/journal.ppat.1006390 (PMC5472306; doi:10.1371/journal.ppat.1006390)
Supplement: S1 Fig — (PDF) [file ppat.1006390.s005.pdf]

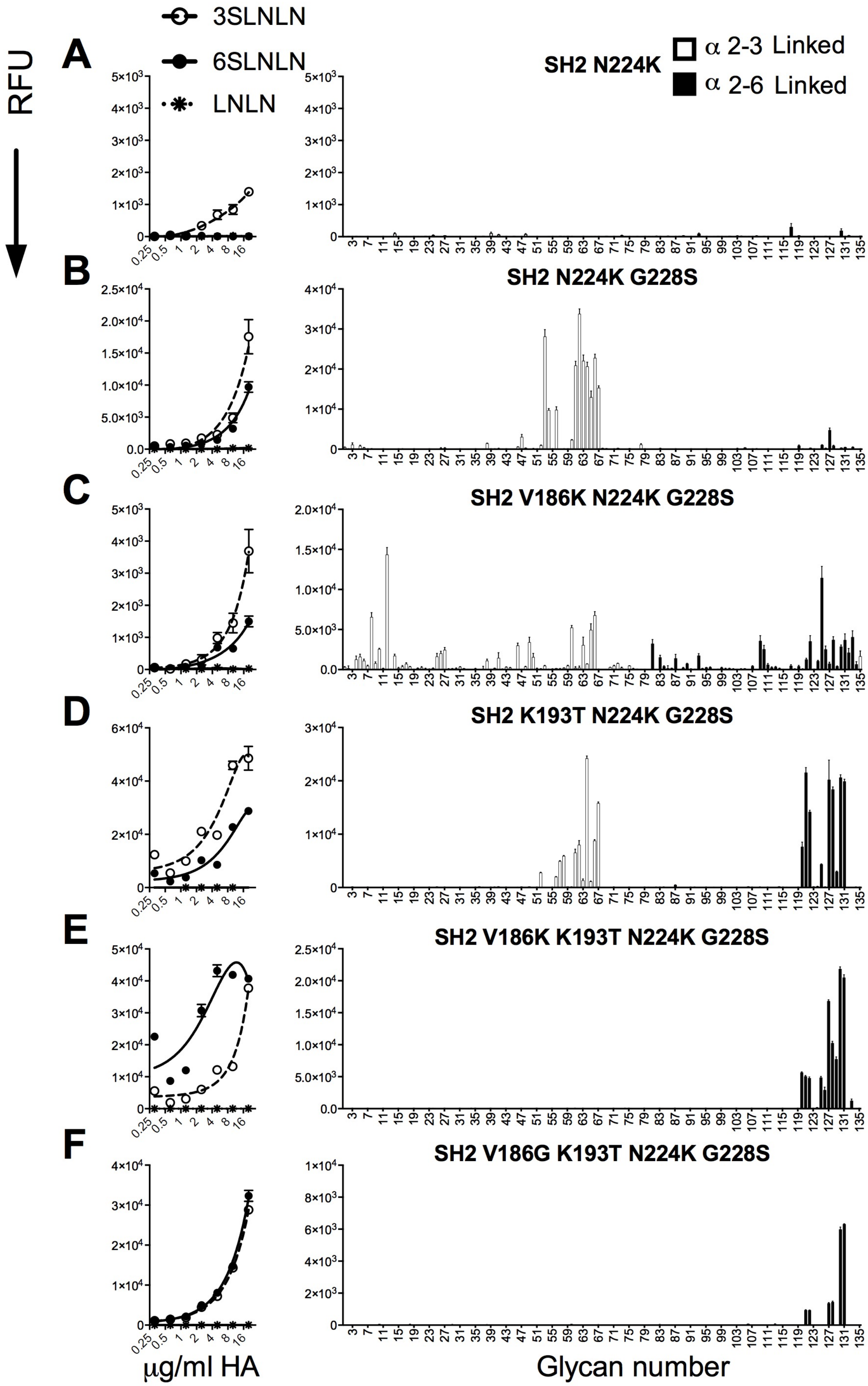

3SLNLN
6SLNLN
LNLN
α 2-3 Linked
α 2-6 Linked
RFU
A
SH2 N224K
B
SH2 N224K G228S
C
SH2 V186K N224K G228S
D
SH2 K193T N224K G228S
E
SH2 V186K K193T N224K G228S
F
SH2 V186G K193T N224K G228S
μg/ml HA
Glycan number

S1 Fig. Receptor binding of SH2 N224K mutants. Glycan binding analyses of Sh2 H7N9 N224K mutant HAs, N224K (A), N224K H228S (B), V186K N224K G228S (C), K193T N224K G228S (D), V186K K193T N224K G228S (E), V186G K193T N224K G228S (F). The mean signal and standard error were calculated from six independent replicates on both the PAA (left column) and the sialoside array (right column). In the PAA array, white open circles represent α2-3 linked sialylated di-LacNAc (3'SLNLN), black closed circles represent α2-6 linked sialylated di-LacNAc (6'SLNLN) and non-sialylated di-LacNAc (LNLN) is represented in asterisks. In the sialoside array, α2-3 linked sialosides are shown in white bars (glycans 11 to 79 on the x axis) and α2-6 linked sialosides in black (glycans 80 to 135). Glycans 1 to 10 are non-sialylated controls (see also S1 Table).
