# Supplementary material for: Three mutations switch H7N9 influenza to human-type receptor specificity
Source: PLoS Pathog. 2017 Jun 15;13(6):e1006390. doi: 10.1371/journal.ppat.1006390 (PMC5472306; doi:10.1371/journal.ppat.1006390)
Supplement: S2 Fig — (PDF) [file ppat.1006390.s006.pdf]

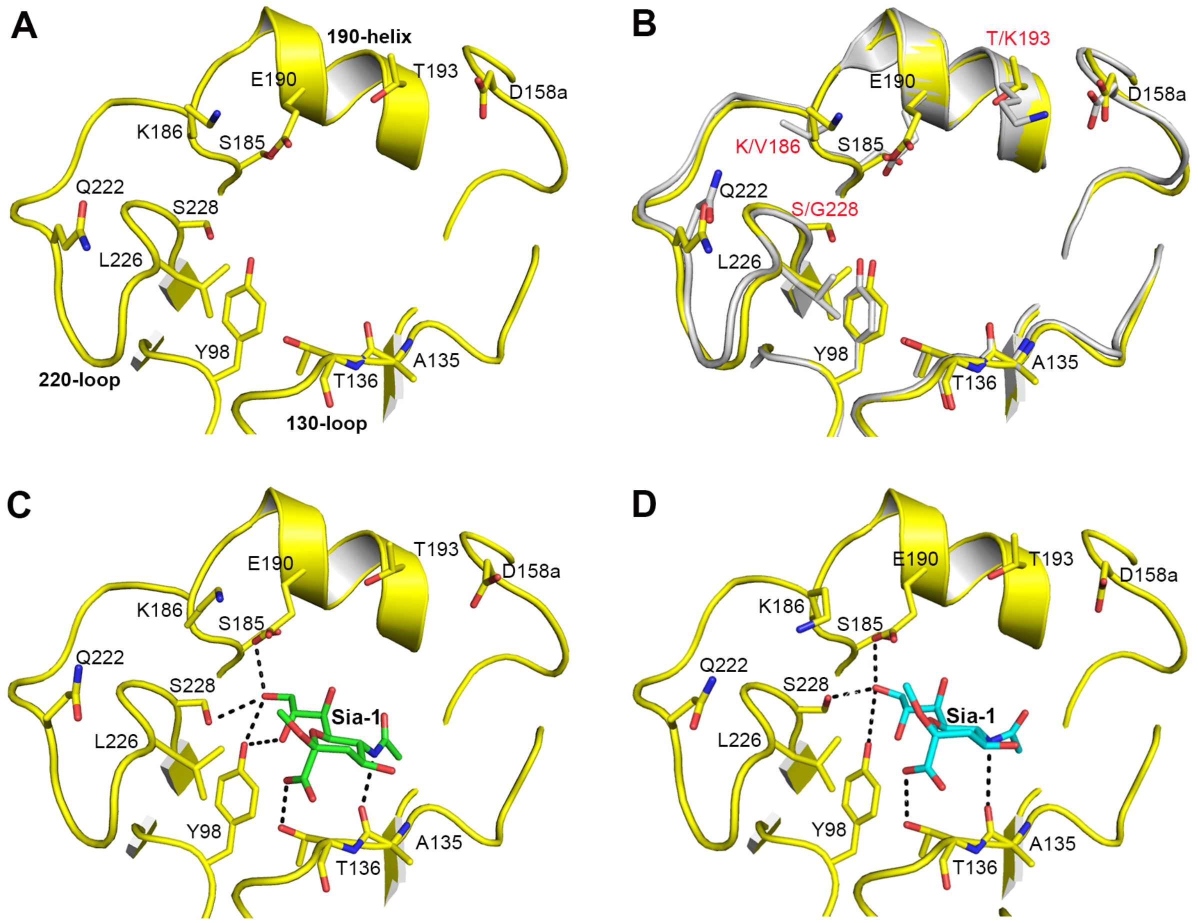

S2 Fig. Crystal structures of human Sh2 H7N9 HA mutants. The receptor binding subdomain (residues 117-265) was used to superimpose the different HA structures for comparison. (A) The receptor binding site (RBS) of the apo-form H7 HA with three mutations G228S, V186K and K193T (yellow tubes for the backbone with selected residues in the binding site shown in atomic representation with yellow carbon atoms, blue nitrogen atoms and red oxygen atoms). (B) Structural comparison of the RBS of Sh2 H7 HA triple mutant (with yellow side chains and C_α atoms) and wild-type Sh2 H7 HA (PDB code 4N5J, with grey side chains and C_α atoms). The overall structures as well as key binding residues are virtually identical except for the side chains of the three mutations and some neighboring side chains, as well as a few minor side-chain differences, such as for L226. The overall C_α RMSD between the structures is 0.45 Å for the receptor binding subdomain. (C) The RBS of the H7 HA triple mutant (yellow tubes for the backbone with selected residues in the binding site in atomic representation with yellow carbon atoms, blue nitrogen atoms and red oxygen atoms) in complex with human receptor analog LSTc (green carbon atoms, blue nitrogen atoms and red oxygen atoms). Only the sialic acid is observed in the electron density maps (S3 Fig). (D) The RBS of the H7 HA triple mutant in complex with avian receptor analog LSTa (cyan carbon atoms, blue nitrogen atoms and red oxygen atoms). Only the sialic acid of the sialoside is observed in the electron density map (S3 Fig).
