# Supplementary material for: Three mutations switch H7N9 influenza to human-type receptor specificity
Source: PLoS Pathog. 2017 Jun 15;13(6):e1006390. doi: 10.1371/journal.ppat.1006390 (PMC5472306; doi:10.1371/journal.ppat.1006390)
Supplement: S3 Fig — (PDF) [file ppat.1006390.s007.pdf]

A B

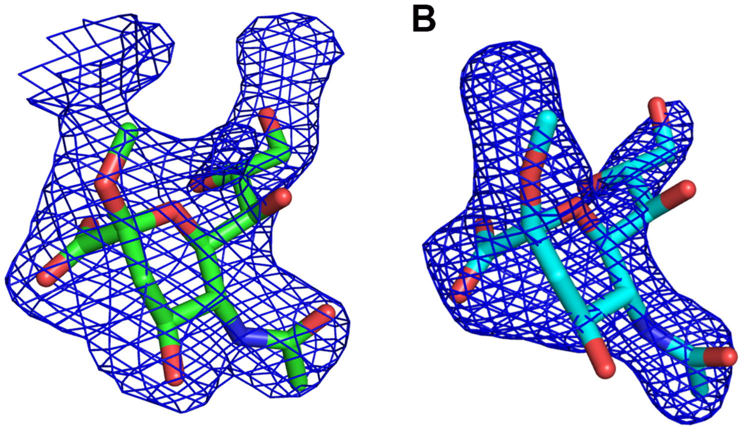

S3 Fig. Simulated annealing omit (Fo-Fc) electron density maps of glycan ligands bound to H7 HA triple mutant. (A) LSTc bound to the triple mutant HA. (B) LSTa bound to the triple mutant HA. The electron density for the glycan receptors is represented in a blue mesh and contoured at 2.5 σ.
